# Gestational Duration and Postnatal Age‐Related Changes in Aperiodic and Periodic Parameters in Neonatal and Toddler Electroencephalogram (EEG)

**DOI:** 10.1002/hbm.70130

**Published:** 2025-01-07

**Authors:** Silja Luotonen, Henry Railo, Henriette Acosta, Minna Huotilainen, Maria Lavonius, Linnea Karlsson, Hasse Karlsson, Jetro J. Tuulari

**Affiliations:** ^1^ FinnBrain Birth Cohort Study, Turku Brain and Mind Center, Department of Clinical Medicine University of Turku Turku Finland; ^2^ Centre for Population Health Research Turku University Hospital and University of Turku Turku Finland; ^3^ Department of Pediatric Neurology Turku University Hospital Turku Finland; ^4^ Department of Clinical Neurophysiology University of Turku Turku Finland; ^5^ Department of Psychiatry and Psychotherapy Philipps University of Marburg Marburg Germany; ^6^ Centre of Excellence in Music, Mind, Body, and Brain, Faculty of Educational Sciences University of Helsinki Helsinki Finland; ^7^ Cognitive Brain Research Unit, Faculty of Medicine University of Helsinki Helsinki Finland; ^8^ Department of Clinical Medicine, Unit of Public Health University of Turku Turku Finland; ^9^ Department of Child Psychiatry Turku University Hospital Turku Finland; ^10^ Department of Psychiatry Turku University Hospital and University of Turku Turku Finland; ^11^ Turku Collegium for Science, Medicine and Technology University of Turku Turku Finland

**Keywords:** aperiodic activity, children, EEG, gestational duration, neonates, SpecParam, spectral parametrization

## Abstract

The brain develops most rapidly during pregnancy and early neonatal months. While prior electrophysiological studies have shown that aperiodic brain activity undergoes changes across infancy to adulthood, the role of gestational duration in aperiodic and periodic activity remains unknown. In this study, we aimed to bridge this gap by examining the associations between gestational duration and aperiodic and periodic activity in the EEG power spectrum in both neonates and toddlers. This cross‐sectional study involved EEG data from 73 neonates (postnatal age 1–5 days, 40 females) and 56 toddlers (postnatal age of 2.9–3.2 years, 28 females) from the FinnBrain Birth Cohort Study. EEG power spectra were parameterized to aperiodic and periodic components using the SpecParam tool. We tested the associations between gestational duration as well as postnatal age and SpecParam parameters in neonates and toddlers while including birth weight and child sex as covariates. For neonates, multilevel models were employed, considering different data acquisitions (sleep and auditory paradigm + sleep), while in toddlers, regression models were used as only data from the auditory paradigm was available. We found that longer gestational duration was associated with a steeper power spectrum across EEG frequencies both in neonates and toddlers. Effect was especially strong in toddlers (*β* = 0.45, *p* = 0.004), while in neonates, it remained nearly statistically significant (*p* = 0.061). In neonates, a quadratic association between gestational duration and beta center frequency (12.5–30 Hz) was found. In toddlers, beta center frequencies were overall higher in females compared to males. Offset (calculated as the power of the aperiodic curve at 2.5 Hz) and theta center frequency had negative associations with postnatal age in neonates, but not in toddlers.

Our results suggest that gestational duration may have significant and relatively long‐lasting effects on brain physiology. The possible behavioral and cognitive consequences of these changes are enticing topics for future research.

## Graphical abstract

This study explores the association between gestational duration and brain electrophysiological activity, using EEG data from neonates and toddlers. Our results indicate that longer gestation correlates with a steeper EEG power spectrum in both neonates and toddlers, suggesting that gestational duration may have significant and relatively long‐lasting effects on brain physiology.

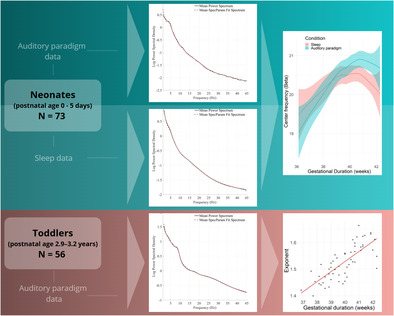

**Summary**

- Longer gestational duration correlates with a steeper power spectrum across EEG frequencies in both neonates and toddlers, with stronger effect observed in toddlers.
- Gestational duration may have significant and relatively long‐lasting effects on brain physiology.
- Our findings suggest sex‐related differences in early brain activity, with female toddlers demonstrating higher beta (12.5–30 Hz) center frequencies compared to their male peers.

## 1 Introduction

Brain development is a dynamic process, with the most rapid growth occurring during gestation and the first postnatal months (Knickmeyer et al. 2008). It involves various maturation processes, including the functional specialization of gray matter and the myelination of white matter, which continues from late gestation into early adulthood (Dubois et al. 2014; Thomason 2020). Especially the prenatal period appears to be particularly critical for neurodevelopment, as disruptions such as premature birth are associated with a range of adverse developmental outcomes (Allen 2008), highlighting the importance of studying gestational duration‐related changes in neurodevelopment.

Prior research of gestational age‐related effects on child neurodevelopment is largely focused on preterm children. Premature birth is a global phenomenon, affecting approximately 11% of births, with about two‐thirds occurring without evident risk factors (Vogel et al. 2018). It is typically defined as birth before the gestational age of 37 weeks (WHO: Recommended Definitions, Terminology and Format for Statistical Tables Related to the Perinatal Period and Use of a New Certificate for Cause of Perinatal Deaths. Modifications Recommended by FIGO as Amended October 14, 1976, 1977). Children born prematurely are known to be at higher risk for various developmental conditions, including cognitive, sensory, language, visual‐perceptual, attention, and learning deficits, as well as cerebral palsy, compared with their full‐term peers (Allen 2008; Taine et al. 2018). However, the binary nature of maturity has been challenged, and a continuum of gestational age‐related effects has been proposed (Engle 2011).

Emerging evidence suggests that even neonates born between gestational weeks 37 and 38, termed early‐term infants, may face adverse outcomes, indicating a continuum of gestational age‐related effects (Dong, Chen, and Yu 2012). These early‐term infants exhibit risks for delayed neurodevelopment, adverse behavioral and emotional outcomes, as well as long‐term social difficulties compared to their term‐born peers (Dong, Chen, and Yu 2012; Wu et al. 2021; Boyle et al. 2012). These effects can be observed as late as school age, with poorer outcomes in school performance noted at the age of 7 years, although not to the extent seen in preterm peers (Chan and Quigley 2014). Conversely, a longer gestational duration in full‐term infants (gestational age > 37 weeks) has been associated with higher scores of standard assessment of mental and motor development during the first year of life (Espel et al. 2014). Consequently, there is a growing need to find methods to study factors that mediate these effects.

Prior research suggests that gestational age may directly influence brain development, thereby mediating the risk for adverse neurodevelopmental outcomes (Engle 2011). For instance, Gale‐Grant et al. (2022) utilized diffusion tensor imaging in term‐born infants and demonstrated lower fractional anisotropy and higher mean, axial, and radial diffusivity in major white matter tracts as a function of gestational age and showed the potential of neuroimaging findings to elucidate the effects of gestational age on infant later neurodevelopment. Similarly, studies in preterm infants have reported smaller brain volumes (Alexander et al. 2019; Inder et al. 2005; Parikh et al. 2013; Thompson et al. 2007; Vasu et al. 2014) and higher diffusivity in various cortical regions, likely reflecting delayed maturation of these areas (Bouyssi‐Kobar et al. 2018). Moreover, preterm infants exhibit alterations in structural networks, particularly in subcortical systems (Zheng et al. 2023), and impairments in functional connectivity (Eyre et al. 2021), potentially underpinning later developmental difficulties. Functional brain maturity of preterm infants can be estimated accurately from EEG, and the results correlate strongly with postmenstrual age (Stevenson et al. 2020). Early postnatal EEG has also been shown to have potential in predicting cognitive outcomes of preterm children, with lower total absolute band powers associated with poorer performance in cognitive assessments (Nordvik et al. 2022). The existing body of research mostly focuses on preterm infants, and studies investigating the neurodevelopment of early‐term‐born neonates are relatively scarce, particularly in later measurement points, emphasizing the need for potential neural markers to identify at‐risk children.

Recent electrophysiological studies have proposed aperiodic and periodic activity as promising potential biomarkers for brain maturational processes. Brain signals can be categorized into periodic (oscillatory) and aperiodic (1/f‐like) properties through spectral parameterization (SpecParam, formerly fooof), utilizing methods such as the SpecParam (Donoghue et al. 2020) tool. Periodic activity is characterized by distinct rhythmic peaks observed at regular intervals, whereas aperiodic activity exhibits a 1/f distribution of signal power across frequencies (Donoghue et al. 2020). Despite its earlier designation as “1/f noise”, emerging evidence supports the notion of aperiodic activity as a distinct signal, highlighting the importance of studying aperiodic and periodic activity separately (Donoghue et al. 2020; Voytek and Knight 2015). This is also supported by prior findings showing that aperiodic activity influences the interpretation of periodic oscillations (Schaworonkow and Voytek 2021).

Aperiodic activity can be defined with two parameters: offset and exponent. The offset indicates the uniform shift in power across the frequencies, likely reflecting neuronal population spiking (Manning et al. 2009). In turn, the exponent describes the slope of the power spectrum (i.e., the distribution of aperiodic power across the frequencies), appearing to reflect underlying synaptic currents, such as excitation–inhibition (E–I) balance (Gao, Peterson, and Voytek 2017). Aperiodic activity offers valuable insights into brain maturational processes as growing recent research suggests a decrease of aperiodic exponent and/or offset as a function of postnatal age in infants, children, adolescents, and adults (Schaworonkow and Voytek 2021; Cellier et al. 2021; Clark et al. 2024; Hill et al. 2022; McSweeney et al. 2023; Rico‐Picó et al. 2023; Tröndle et al. 2022). Also, a quadratic effect of postnatal aging on aperiodic exponent and offset between the ages of 4–11 years (McSweeney et al. 2023) as well as findings of increasing aperiodic exponent during the first year of life have been reported (Wilkinson et al. 2024a). However, to the best of our knowledge, changes in aperiodic activity related to gestational age remain unexplored.

This cross‐sectional study investigated gestational duration‐related changes in aperiodic and periodic activity in the EEG power spectrum, both in neonates and toddlers. Additionally, we explored the links between aperiodic and periodic activity and offspring postnatal age while considering birth weight and child sex as covariates. Based on prior electrophysiological studies of age‐related changes in aperiodic activity (Schaworonkow and Voytek 2021; Cellier et al. 2021; Clark et al. 2024; Hill et al. 2022; McSweeney et al. 2023; Rico‐Picó et al. 2023; Tröndle et al. 2022), we hypothesized that gestational duration and postnatal ages of participants are negatively associated with aperiodic parameters, reflecting the maturation processes of the brain during aging.

## 2 Materials and Methods

The aim of the FinnBrain study is to investigate the effects of early‐life stress on child brain development by combining multidisciplinary approaches such as neuropsychological studies, neuroimaging, and molecular genetic studies. The FinnBrain Birth Cohort Study and this sub‐study have been approved by the Ethics Committee of the Hospital District of Southwest Finland (ETMK: 57/180/2011, 15.3.2016§109), and the studies were conducted according to the Declaration of Helsinki.

### 2.1 Participants

Participants of this cross‐sectional study were part of the FinnBrain Birth Cohort Study (Karlsson et al. 2018), a population‐based pregnancy cohort with 3808 families recruited during the years 2011–2015 at the first trimester ultrasound. The sample of the current study included 158 neonates and 123 toddlers born between 2013 and 2015. Neonates were recruited for an EEG recording at the maternity wards of Turku University Hospital on average 1–2 days after birth. Toddlers were recruited during the years 2016–2018. A written informed consent was collected from parents for their offspring to participate in the EEG recordings.

During the initial phase of toddler data collection, we experienced technical issues with the amplifier, resulting in poor‐quality data. To ensure the accuracy and validity of the study, we decided to exclude all data (*N* = 53) collected prior to servicing the amplifier. From the neonate data, one participant was excluded from the study due to missing information of postnatal age.

**FIGURE 1:**
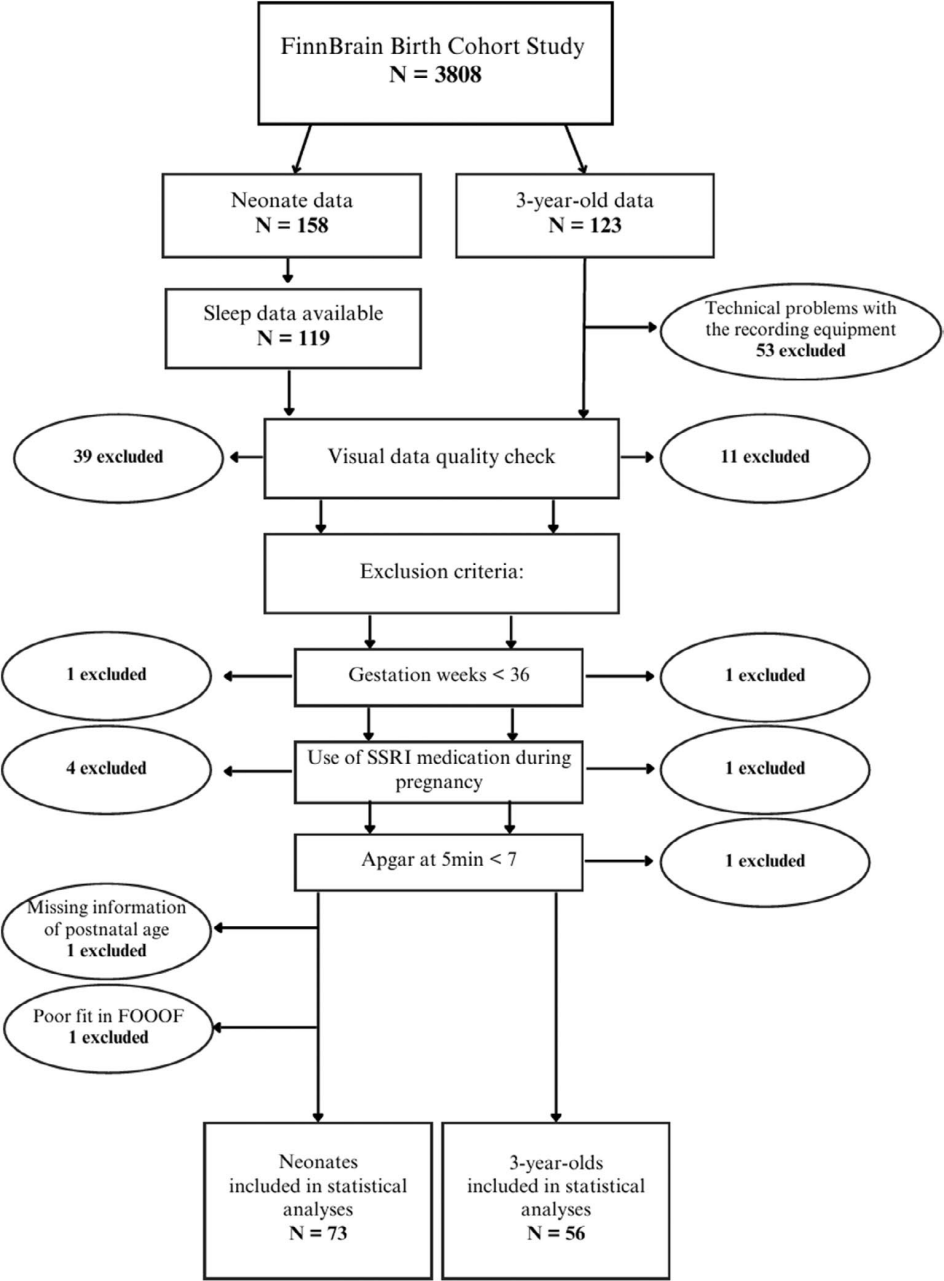
Study flow chart.

Exclusion criteria for this study were: gestational age less than 36 weeks (in neonates *N* = 1, in toddlers *N* = 1), maternal use of selective serotonin reuptake inhibitors (SSRIs) during the pregnancy (in neonates *N* = 4, in toddlers *N* = 1), low (< 7) Apgar score at 5 min after birth (in neonates *N* = 0, in toddlers *N* = 1), and low birth weight (< 1800 g, no excluded participants). One neonate was excluded due to poor fit of SpecParam. Finally, data of 73 neonates and 56 toddlers were used in further statistical analyses (for the flow chart, see Figure 1).

The characteristic variables for neonates, toddlers, and their mothers are presented in Table 1.

**TABLE 1:** Characteristics of participants included in analyses (73 neonates and 56 toddlers) and their mothers.

	*N*	Missing	%	Mean	SD	Range
Neonates						
Gestational weeks (gwk) at birth	73	0		40.04	1.297	36.14–42.29
Sex	73	0				
Female	40		54.80			
Male	33		45.20			
Apgar (5 min)	73	0		9.15	0.49	8–10
Birth weight (g)	73	0		3549.90	476.26	2740.00–5470.00
Birth height (cm)	73	0		50.44	2.18	46–57.00
Postmenstrual age at EEG recording (days)	73	0		281.58	8.77	255.00–297.00
Postnatal age at EEG recording (days)	73	0		1.47	1.11	0–5
Mothers of neonates						
Age at delivery (years)	73	0		30.61	4.27	22–42
Pre‐pregnancy BMI (kg/m2)	73	0		23.45	3.28	17.09–36.42
Native language	67	6				
Finnish	64		95.52			
Swedish	3		4.48			
Educational level (gwk 12)	67	6				
Matriculation examination or lower	28		41.79			
Higher vocational training	18		26.87			
University degree or higher	21		31.34			
Monthly income (gwk 12)	67	6				
< 500 €	5		7.46			
501–1000 €	9		13.43			
1001–1500 €	14		20.90			
1501–2000 €	27		40.30			
2001–2500 €	7		10.45			
2501–3000 €	1		1.49			
3001–3500 €	3		4.48			
3501–4000 €	1		1.49			
Alcohol use during pregnancy (gwk 12)	67	6				
Yes	17		25.37			
No	50		75.63			
Drug use during pregnancy (gwk 12)	67	6				
Yes	1		1.49			
No	66		98.51			
Tobacco smoking during pregnancy (gwk 12)	66	7				
Yes	11		16.67			
No	55		83.33			
SSRI/SNRI use during pregnancy (gwk 12 and gwk 36)	69	4				
No	69		100.00			
Marital status	73	0				
Married	36		49.32			
Unmarried	35		47.95			
Divorced	1		1.37			
Registered partnership	1		1.37			
Toddlers						
Gestational weeks (gwk) at birth	56	0		39.90	1.32	36.86–42.29
Sex	56	0				
Female	28		50.00			
Male	28		50.00			
Apgar (5 min)	56	0		9.02	0.62	7–10
Birth weight (g)	56	0		3626.63	529.06	2600.00–5470.00
Birth height (cm)	55	1		50.66	2.24	46.00–57.00
Postnatal age at EEG recording (months)	56	0		36.66	1.01	34.75–38.17
Daycare status (at the age of 2 years)	35	21				
At home	11		31.43			
Family day care	9		25.71			
Nursery school	13		37.14			
Shift nursery school	1		2.86			
Other	1		2.86			
Number of siblings (at the age of 4 years)^a^	33	23				
None	12		36.36			
One	20		60.61			
Two	1		3.03			
Mothers of toddlers						
Age at delivery (years)	56	0		30.80	4.28	19.00–39.00
Pre‐pregnancy BMI (kg/m^2^)	56	0		24.73	4.89	19.15–36.96
Native language	55	1				
Finnish	54		98.18			
Swedish	1		1.82			
Educational level (gwk 12)	55	1				
Matriculation examination or lower	11		20.00			
Higher vocational training	21		38.18			
University degree or higher	23		41.82			
Monthly income (gwk 12)	55	1				
< 500 €	7		12.73			
501–1000 €	4		7.27			
1001–1500 €	8		14.55			
1501–2000 €	23		41.82			
2001–2500 €	10		18.18			
2501–3000 €	3		5.46			
3001–3500 €	7		12.73			
3501–4000 €	4		7.27			
Alcohol use during pregnancy (gwk 12)	51	5				
Yes	9		17.65			
No	42		82.35			
Drug use during pregnancy (gwk 12)	51	5				
No	51		100.00			
Tobacco smoking during pregnancy (gwk 12)	51	5				
Yes	4		7.84			
No	47		92.16			
SSRI/SNRI use during pregnancy (gwk 12 and gwk 36)	55	1				
No	55		100.00			
Marital status	56	0				
Married	28		50.00			
Unmarried	27		48.21			
Divorced	1		1.79			

^a^ Half‐siblings and different parent siblings also included.

### 2.2 Demographics

The Finnish Medical Birth Register kept by the Finnish National Institute for Health and Welfare (www.thl.fi) was used to provide information on maternal age at delivery (years), pre‐pregnancy body mass index (BMI; kg/m^2^), medication usage during pregnancy, marital status, gestational duration (weeks), neonate birth weight (grams), birth height (cm), Apgar points, and child sex.

### 2.3 EEG Data Collection

In both neonate and toddler measurements, the EEG of the children was recorded using an actiCAP electrode cap (EASYCAP, Germany) and a BrainVision Quickamp amplifier (Brain Products, Germany). EEG was recorded with 16 channels in neonates and 32 electrodes in toddlers. The electrode locations followed the international 10–20 system. The original sampling frequency was 500 Hz in neonates and 250 Hz in toddlers. To achieve analogous sampling frequencies for both datasets for further analyses, neonate EEG data were downsampled to 250 Hz during EEG preprocessing. The reference and ground electrodes were located on the forehead.

#### 2.3.1 Neonates

Most of the EEG measurements were organized in the afternoon (~75%). After the electrode preparation process, if necessary, the neonate was assisted to sleep by offering a pacifier or mild glucose solution. To achieve a calm sleep phase and minimize restlessness, the EEG measurement was conducted within 2 h after feeding the neonate. The alertness of the neonate was visually assessed by the researcher, and if prolonged crying occurred at any point during the recording, the recording session was terminated immediately, ensuring that neonates were sleeping during the recordings.

During the recording, three types of data were collected in the following order: (1) auditory paradigm + sleep data, featuring three types of emotionally valenced pseudo‐words (duration ~12 min; for the fully detailed presentation of the multi‐feature paradigm, see: Kostilainen et al. (2018)), (2) pseudo‐word paradigm data (duration ~14 min), and (3) sleep data (duration ~0–20 min). Sleep data were recorded only if the neonate was still sleeping at the end of other blocks. Because of this, sleep data were not successfully recorded from all participants (~75% of neonates with both auditory paradigm + sleep and sleep alone data recorded). Neonates were positioned on their left side, with the right ear facing upwards. To present the emotionally valenced auditory stimuli, the paradigm was presented twice from a loudspeaker situated approximately 1 m from the neonate at a standard volume of 60 dB. Total duration of the measurement session was approximately 45 min.

#### 2.3.2 Toddlers

Prior to EEG recording, each family received an email containing a brief story introducing the agenda of the visit, along with a couple of pictures for the child to view. This aimed to familiarize the family, especially the child, with the upcoming visit. Measurement visits were organized prioritizing family schedules, including both weekday and weekend as well as morning and afternoon visits. At the beginning of the visit, a teddy bear wearing the electrode cap was used as a visual aid, introducing the preparation steps and creating a comfortable atmosphere before initiating the recording. During the approximately 15‐min electrode preparation process, the child had the opportunity to watch a self‐selected animated series.

In toddlers, differently from neonates, only the auditory paradigm data were recorded. The child was seated either on a chair or on their mother's lap, with the stimuli presented from a loudspeaker located 1.25 m away at a volume of 60 dB. The duration of one auditory paradigm block was approximately 5 min, and it was run twice for each child. A room divider between the child and the investigator helped minimize potential distractions. There was an option for a short break between recording sessions if needed. In order to enhance compliance and to minimize motion artifacts, the children viewed a silent video during the EEG recording (Inscapes movie (Vanderwal et al. 2015) featuring abstract shapes, originally designed to improve compliance in functional MRI). Additional comfort measures, such as toys and drawing tools, were provided only if necessary. Total duration of the measurement session was approximately 45 min.

#### 2.3.3 Auditory Paradigm

During the toddler and neonate EEG recording, the multifeature mismatch negativity paradigm (Näätänen et al. 2004), further developed by adding emotional stimuli variants (Pakarinen et al. 2014), was used. The paradigm includes a standard stimulus, featuring a bisyllabic pseudo‐word (/ta‐ta/) and four distinct types of linguistically relevant deviant stimuli: vowel duration change (/ta‐ta:/ [11% probability, 50 trials in each block], vowel change [/ta‐to/, 11% probability, 50 trials in each block], intensity changes [±6 dB, 5% probability each, 22 trials in block], and frequency changes [±25.5 Hz, 6% probability each, 28 trials per block]) In addition to that, three emotionally loaded uttered stimuli (angry, sad, and happy variants of /ta‐ta/) were presented with durations of 388, 337, and 436 ms, respectively, each occurring with a 3% probability across 12 trials per block for each emotion. The duration of the standard stimulus is 336 ms, presented with a 46% probability across 200 trials per block. For the more detailed presentation of the employed experiment, see: Kostilainen et al. (2018) and Lavonius et al. (2020).

### 2.4 Preprocessing

First, EEG data from both neonates and toddlers were visually assessed, and datasets showing consistently poor data quality were excluded (in neonates, *N* = 39; in toddlers, *N* = 11). Second, the artifact subspace reconstruction (ASR) method (Kothe and Jung 2014) was applied using the *clean_artifacts* function of MATLAB (r2018b, version 9.5.0, The MathWorks Inc.) with default settings to preprocess the data. ASR is an automatic artifact removal method that employs statistical and machine learning techniques to identify, model, and eliminate artifacts (Chang et al. 2020). It is designed for selective artifact removal while striving to preserve the underlying neural signals (Chang et al. 2020).

To summarize, ASR includes the following steps: First, it filters the data with a high‐pass filter (transition band [0.25 0.75] Hz) and detects flat channels (longer than 5 s) and poorly correlated channels (minimum correlation threshold of 0.85). The algorithm identifies artifact segments and estimates the subspace of the data. A threshold is then applied to determine segments that deviate significantly from the estimated subspace (with a cutoff for high amplitude bursts at 5 SD). Segments that are deemed uncoverable as well as channels consistently identified as problematic are removed. The algorithm reconstructs the remaining segments using only the components of the data that align with the estimated subspace.

After that, channels removed during ASR were interpolated. Information on interpolated channels is presented in Table S1. The data was re‐referenced to a common average reference. Mean power spectra across participants of each data set showing each channel separately were visually assessed before and after the preprocessing to ensure the data quality was acceptable (Figure S1).

### 2.5 Parameterization of the Power Spectrum

The power spectrum calculations and SpecParam were done separately for the three data sets: the neonate sleep data, the neonate auditory paradigm + sleep data, and the toddler auditory paradigm data.

Prior to calculating the power spectrum, we followed Schaworonkow and Voytek (2021) by dividing the continuous EEG data of each participant and channel into segments of 30 s, aiming to increase the reliability of calculating the parameters of the power spectrum. Power spectral density (PSD) was calculated using Welch's method for every segment (window length 500 samples, 50% overlap). Finally, the Python‐based SpecParam toolbox (Donoghue et al. 2020) was employed to parameterize the spectral data by separating the aperiodic and periodic components of the signal.

In SpecParam, the aperiodic signal, *L*, is modeled as:

$$\text{34𝐿}=\text{34𝑏}-\log\left({\text{34𝐹}}^{x}\right)$$

with *b* determining the aperiodic offset and x determining its slope (aperiodic exponent) when plotted on a log–log axis.

For the present study, we parameterized the spectra in the 1–45 Hz frequency range to cover the canonical (M)EEG frequency bands from theta to beta (Pernet et al. 2020). To assess whether the data has any periodic peaks around 45 Hz that could impact our aperiodic fit, we provided mean and individual plots of power spectra from 0 to 50 Hz. To avoid the 50 Hz spike related to line frequency, we set the upper frequency boundary to 45 Hz to ensure that we were analyzing pure electrophysiological data (Figure S2). SpecParam settings used in this study mainly followed the prior study on infants by Schaworonkow and Voytek (2021). The settings were: peak width limits = [1.5, 12.0], maximum number of peaks = 5, peak threshold = 2.0, minimum peak height = 0.0, and aperiodic mode: “fixed.”

From the SpecParam outputs, the parameters (offset, exponent, and center frequencies from fitted oscillations) from model fits with a minimum *R* ^2^ value of 0.95 were kept for further analyses (*N* = 1 neonate excluded). If the model fit of a segment did not meet the criteria, it was excluded from further analyses. Model fit *R* ^2^'s, number of segments, and number of poor‐fit segments per channel are presented in Table S2, and histograms of model fit *R* ^2^'s as well as errors are presented in Figure S3. We also followed Ostlund et al. (2022) by reporting the absolute errors of the SpecParam fit (Figure S4). As the absolute errors were higher near the frequency boundaries, we ended up calculating the offset values as the power of an aperiodic curve at 2.5 Hz, following Wilkinson et al. (2024b). Finally, the parameter values were averaged over the segments, resulting in one parameter (offset, exponent, or center frequencies [theta/alpha/beta]) value per electrode for each participant.

**FIGURE 2:**
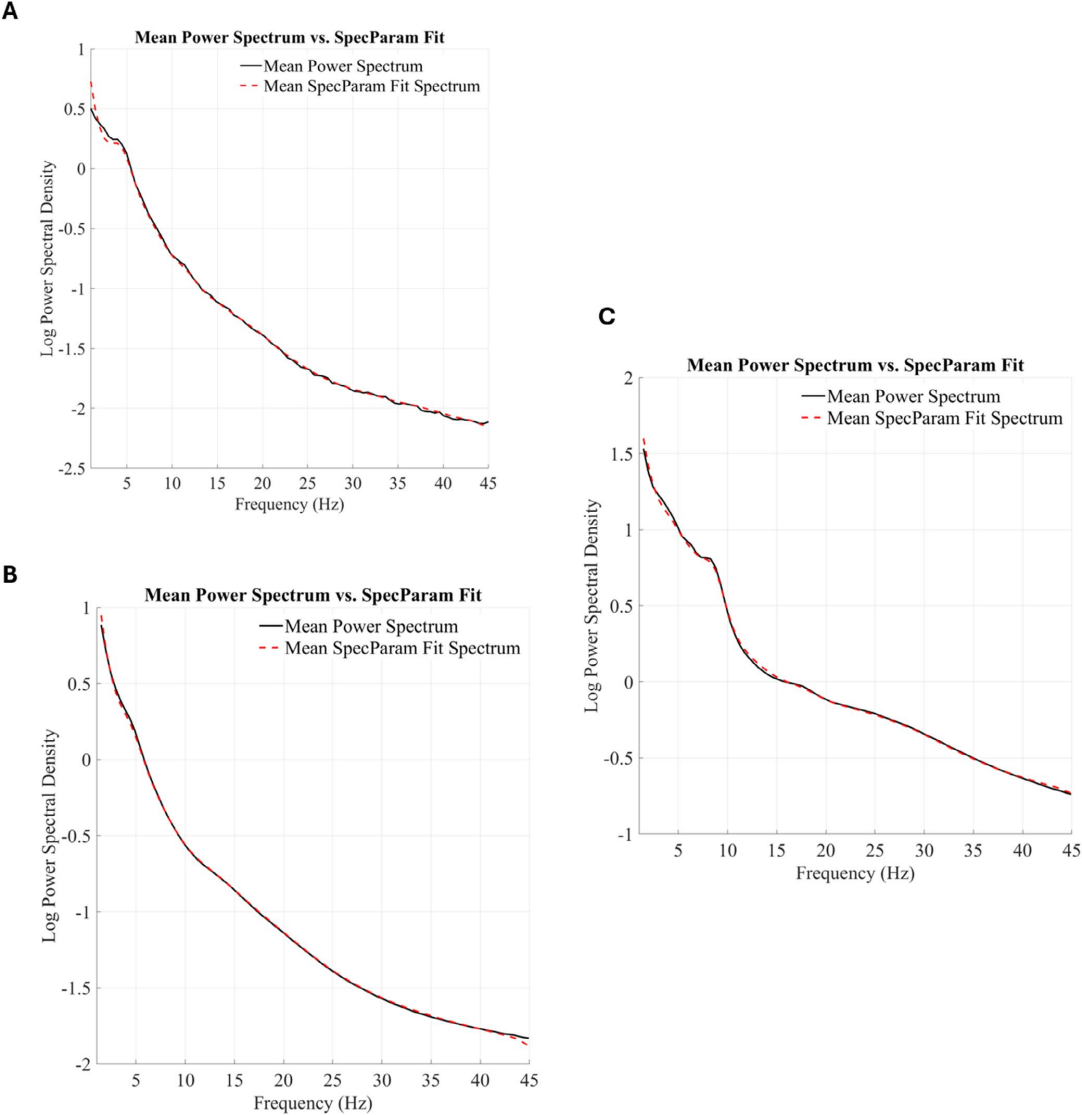
Mean power spectrum versus mean SpecParam fit spectrum (averaged across segments, electrodes, and subjects). (A) Neonates/sleep (*N* = 73). (B) Neonates/auditory paradigm + sleep (*N* = 73). (C) Toddlers/auditory paradigm (*N* = 56).

To see an example of SpecParam model fits versus the original power spectrum averaged across subjects in each of the three data sets, see Figure 2.

### 2.6 Aperiodic Parameters (Exponent and Offset)

**FIGURE 3:**
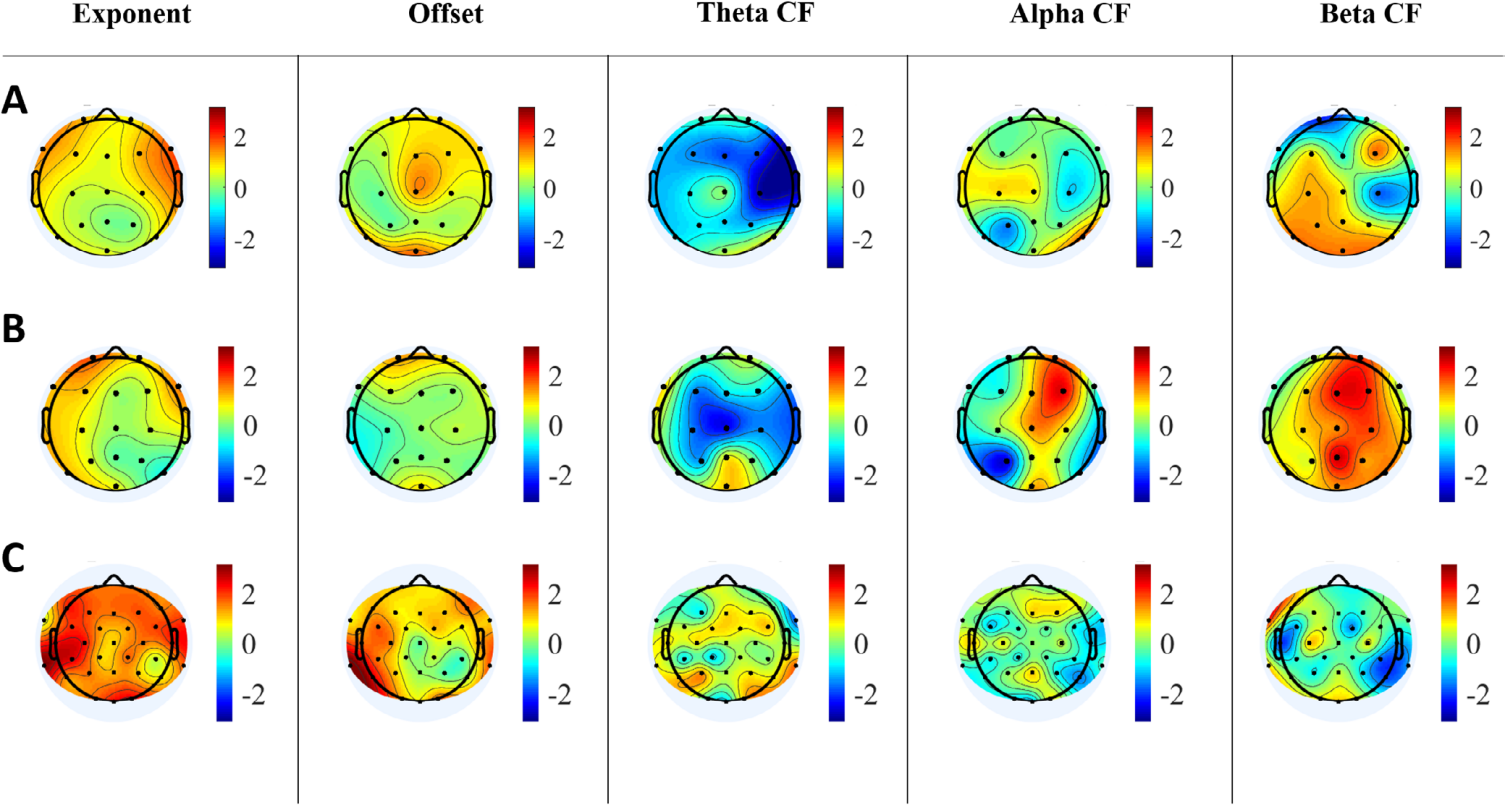
T‐values of correlations between gestational duration and aperiodic/periodic parameters of all EEG electrodes. Red color indicates positive and blue color negative associations. (A) Neonate sleep data. (B) Neonate auditory paradigm + sleep data. (C) Toddler auditory paradigm data. CF = center frequency.

First, we visually inspected the correlations between the gestational duration (weeks) and the aperiodic parameters in both the neonate and toddler datasets. As these revealed a global positive correlation between age variables and the exponent across the scalp in the toddler dataset (Figure 3), we followed Hill et al. (2022) and Stanyard et al. (2024) by averaging the data across the electrodes, resulting in a “global” exponent and offset value reflecting the mean signal across the scalp. By doing this, we also aimed to avoid the problem of multiple comparisons.

### 2.7 Periodic Parameters (Center Frequency)

**FIGURE 4:**
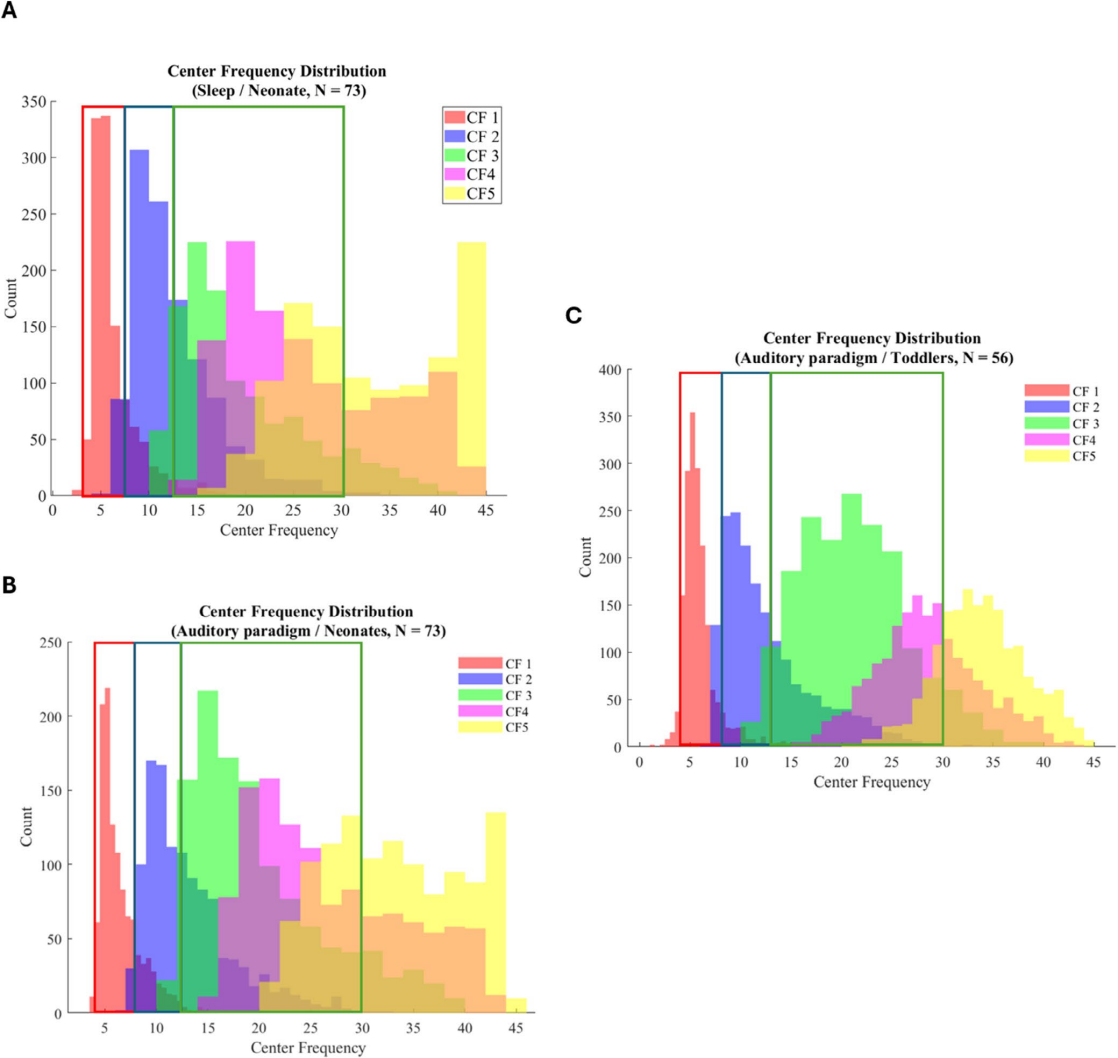
Center frequency (CF) distributions of SpecParam‐fitted oscillations in each data set. Selected frequency bins are marked with boxes (red = 4–8 Hz (theta)/blue = 8–12.5 Hz (alpha)/green = 12.5–30 Hz (beta)). (A) Sleep/neonates (*N* = 73). (B) Auditory paradigm + sleep/neonates (*N* = 73). (C) Auditory paradigm/toddlers (*N* = 56).

The center frequency peak parameters were extracted from the periodic signal for selected frequency bins (theta: 4–8 Hz, alpha: 8–12.5 Hz, beta: 12.5–30 Hz). These frequency bins were selected based on visual inspection of the distribution of center frequency values of the fitted oscillations from SpecParam (Figure 4), and they are also very closely corresponding to recommended frequency bands to use in (M)EEG research by Pernet et al. (2020). Because of overlapping oscillations within the selected frequency bins, we calculated the mean value for each bin by averaging the fitted center frequencies within that frequency bin. Visual inspection of age parameters and center frequency values was done similarly to aperiodic parameters, and abroad negative correlation between postmenstrual age and theta center frequency across electrodes was revealed in neonates (Figure 3). Based on this, also the center frequency values were averaged across the electrodes, resulting in “global” center frequency values for each of the frequency bins (theta, alpha, beta) reflecting the mean signal across the scalp.

### 2.8 Statistical Analyses

In this study, we utilized different statistical models due to the structure of each dataset. For the toddlers, who were assessed under a single condition with one measurement per individual, we performed a standard linear regression model due to the independence of observations. Conversely, the neonates were measured under two distinct conditions (sleep/auditory paradigm + sleep), resulting in multiple, correlated observations per individual. To address this, we employed multilevel linear regression models, providing more precise and valid inferences about the effects of the condition. We report raw *p*‐values throughout the manuscript.

#### 2.8.1 Multilevel Models for Neonates

Statistical analyses were performed using RStudio (version 2023.12.0.369) (R Core Team 2022) and JASP (2022, version 0.16.3) (JASP 2022). To study the relationship between aperiodic and periodic parameters and age, we employed multilevel models (MLMs) from the *nlme* package (Pinheiro et al. 2007) in RStudio. The maximum likelihood estimation was used in the MLMs. The models primarily had the following structure:

$$\begin{array}{c} \text{Aperiodic}/\text{periodic parameter}\sim \mathrm{GD}+\mathrm{Age}+\mathrm{Sex}\\ +\text{Birth weight}+\text{Condition}+\left(1|\mathrm{ID}\right) \end{array}$$

where aperiodic parameter means either offset or exponent and periodic parameter means center frequency value from frequency bins (theta, alpha, beta). GD stands for gestational duration (weeks; z‐transformed), while age means the postnatal age of the neonate at the EEG recording (days; z‐transformed). Sex means the biological sex of the child (male/female; categorical variable). Birth weight is the weight of the child after birth (kilograms; z‐transformed). Condition means the paradigm during the EEG data recording (auditory paradigm + sleep/sleep data; categorical variable).

The models were performed separately for each outcome variable (exponent, offset, or center frequencies). The fixed effects were chosen for models by their theoretical relevance and following previous studies (McSweeney et al. 2023). The independent variables were chosen by adding them into the model one by one. Added variables generally decreased Akaike information criterion (AIC) of at least one of the neonate models, except child sex, which increased AIC slightly in all models. As we wanted to keep neonate and toddler models congruent, sex was decided to be included in the final models. The random‐effect structure of the model included separate intercepts for participants. To control the possible effect of the exponent on offset values, the aperiodic exponent (z‐transformed) was included as an independent variable in the offset model.

Following McSweeney et al. (2023), we also formed quadratic models that included the square of gestational duration as a predictor. When comparing the quadratic and linear models, the quadratic models fitted the data significantly better compared to the linear models only in the beta center frequency model (L.ratio = 4.62, *p* = 0.03). For other outcome variables, linear models seemed to fit the data significantly better (exponent: L.ratio = 0.65, *p* = 0.42; offset: L.ratio = 1.81, *p* = 0.18; center frequency (theta): L.ratio = 0.41, *p* = 0.52; center frequency (alpha): L.ratio = 0.53, *p* = 0.47). Considering this, we report the results of the quadratic model for the beta center frequency and the results of the linear models for the other outcome variables in the Results section.

We also included the interaction terms of condition and GD/Age/Sex/Birth weight as fixed effects into the model one by one. Only the interaction effect between Condition and GD in the beta center frequency model was statistically significant (*p* = 0.049). Otherwise, no statistically significant interaction effects were found, and AIC values of the other models also increased (compared to the model with only the main effects), so the interaction terms were not included in the other models.

Residual outliers (± 3 SD) were excluded from the models. The number of residual outliers excluded from each of the models was as follows: exponent (*N* = 3), offset (*N* = 2), center frequency/theta (*N* = 0), center frequency/alpha (*N* = 1), and center frequency/beta (*N* = 0). Furthermore, the model assumptions were visually assessed for every model. Residual versus fitted plots, histograms of the residual terms, and Q–Q plots were visually inspected and estimated acceptable.

Finally, as sensitivity analyses, the maternal pre‐pregnancy BMI (kg/m^2^), maternal tobacco smoking (at the first trimester of pregnancy), and postmenstrual age (PMA, i.e., gestational duration + postnatal age) were included as fixed effects in the model one by one. Model diagnostics remained acceptable, and no statistically significant effects of BMI, tobacco smoking, or postmenstrual age were found.

To further explore whether PMA or gestational duration better explains the outcome variable, we conducted stepwise regression models separately with the original models (using gestational duration as the independent variable) and modified models (in which gestational duration was replaced with PMA). We did not include gestational duration and PMA into the same model because of the multicollinearity issues. In the models in which gestational duration was a significant predictor, PMA was also a significant predictor. However, the *R* ^2^'s of the models were higher in original models using gestational duration as the independent variable, suggesting that gestational duration was a better predictor of the outcome variable.

**FIGURE 5:**
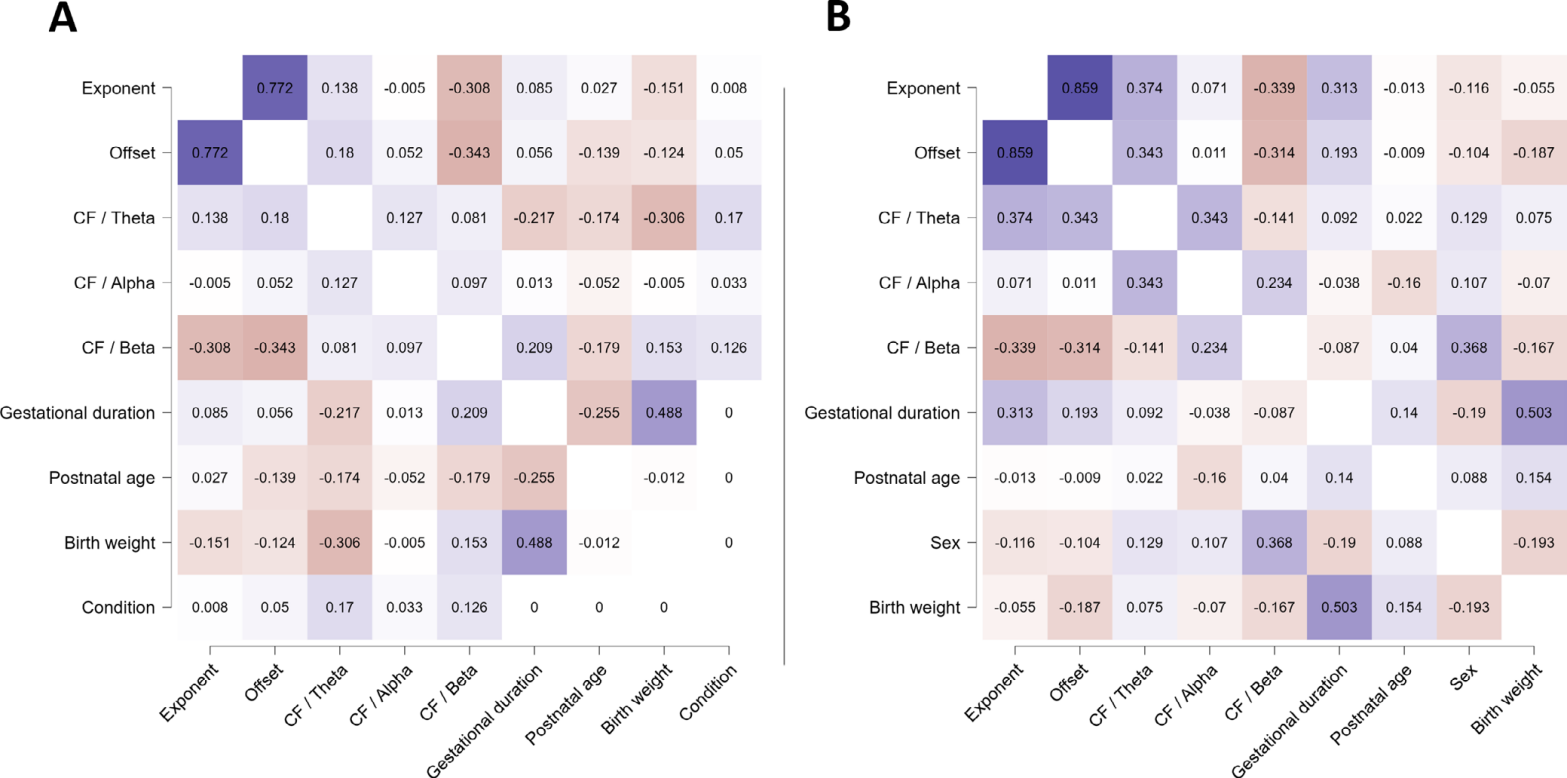
Correlation matrix (Spearman correlation) of the model variables. (A) Neonates. (B) Toddlers. Purple color indicates positive and red color negative correlations.

For the correlation matrix of model variables, see Figure 5.

#### 2.8.2 Linear Regression Models for Toddlers

Statistical analyses were performed using RStudio (version 2023.12.0.369) (R Core Team 2022) and JASP (2022, version 0.16.3) (JASP 2022). To study the associations between the aperiodic (exponent or offset) or periodic parameters (center frequency from theta/alpha/beta frequency band) and age, the *lm* function was used. The linear regression models had the following structure:

Aperiodic/periodic parameter~GD+Age+Sex+Birth weight

where aperiodic parameter means either offset or exponent and periodic parameter center frequency of selected frequency bands (theta/alpha/beta). GD stands for gestational duration (weeks; z‐transformed). Age means the postnatal age (age from birth) at the EEG measurement (months; z‐transformed). Sex means the biological sex of the child (categorical variable). Birth weight is the weight of the child after birth (kilograms; z‐transformed).

The dependent variable in the linear regression models was either offset, exponent, or center frequency (theta/alpha/beta). The independent variables were chosen, similarly to the neonate model, by adding them into the model one by one. Adding birth weight decreased the AIC values of exponent and offset models, supporting their inclusion in the model. To control the possible effect of exponent on offset values, the aperiodic exponent (z‐transformed) was included as an independent variable in the offset model.

The model assumptions were assessed. The variance inflation factors (VIFs) of the models were low (< 1.61 in all models). The residuals were normally distributed (Shapiro–Wilk *p* = 0.71/0.20/0.04/0.61/0.84). While the *p*‐value of the Shapiro Wilk's test was < 0.05 in the theta center frequency model, the visual inspection of the residual histogram supported the normal distribution. Model diagnostics were visually inspected and considered acceptable. Correlations of the residuals were > 0.98 in each model. There were no residual outliers (defined as values greater than +3 SD or less than −3 SD from the mean) to exclude in any of these models.

Finally, we conducted sensitivity analyses to explore potential associations between maternal pre‐pregnancy BMI (kg/m^2^), maternal tobacco smoking (during the first trimester of pregnancy), and postmenstrual age (i.e., gestational duration + postnatal age) with aperiodic or periodic parameters. Each variable was included as an independent variable in separate models. Model diagnostics remained acceptable, and no statistically significant effects of BMI, tobacco smoking, or postmenstrual age were found.

To further explore whether PMA or gestational duration better explains the outcome variable, we conducted stepwise regression models separately with original models (using gestational duration as the independent variable) and modified models (in which gestational duration was replaced with PMA). We did not include gestational duration and PMA into the same model because of the multicollinearity issues. In the models in which gestational duration was a significant predictor, PMA was also a significant predictor. However, the *R* ^2^'s of the models were higher in the original models using gestational duration as the independent variable, suggesting that gestational duration was a better predictor of the outcome variable.

For the correlation matrix of model variables, see Figure 5.

## 3 Results

### 3.1 Neonates

#### 3.1.1 Exponent

**FIGURE 6:**
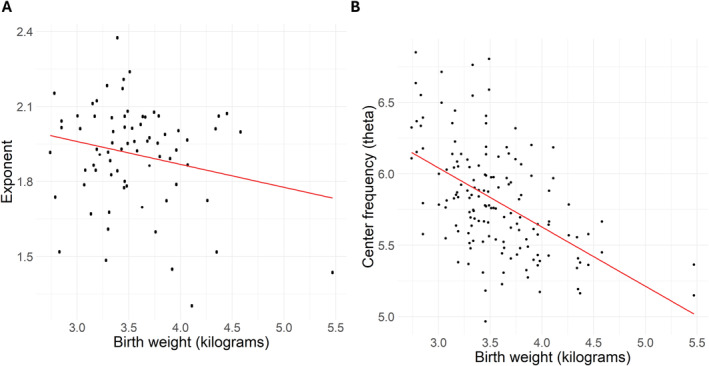
Scatterplot showing the relationship between birth weight (kilograms) and the predicted exponent (A) and theta center frequency (B) values, with a linear regression line fitted to the predicted data points.

The association between gestational duration and exponent was nearly statistically significant (Estimate = 0.06, SE = 0.03, *p* = 0.061), suggesting that the exponent increased as a function of gestational duration. In addition, an increase of one standard deviation (SD) in birth weight of the neonate predicted a statistically significant 0.07 unit decrease in the exponent (Estimate = −0.07, SE = 0.03, *p* = 0.022). For the results, see: Table 2 and Figure 6A.

**TABLE 2:** Results of the multilevel model for exponent in neonates (*N* = 73/observations = 143). Residual outliers (*N* = 3) excluded.

	Estimate	SE	DF	*t*	*p*
Intercept	1.92	0.04	69	45.64	**< 0.001**
Gestational duration	0.06	0.03	68	1.91	0.061
Postnatal age	0.02	0.03	68	0.58	0.561
Condition	0.00	0.02	69	−0.19	0.852
Sex	−0.01	0.06	68	−0.17	0.865
Birth weight	−0.07	0.03	68	−2.34	**0.022**
Marginal *R* ^2^/conditional *R* ^2^ of the model					0.074/0.757

*Note:* Statistically significant *p*‐values (< 0.05) are highlighted.

#### 3.1.2 Offset

**FIGURE 7:**
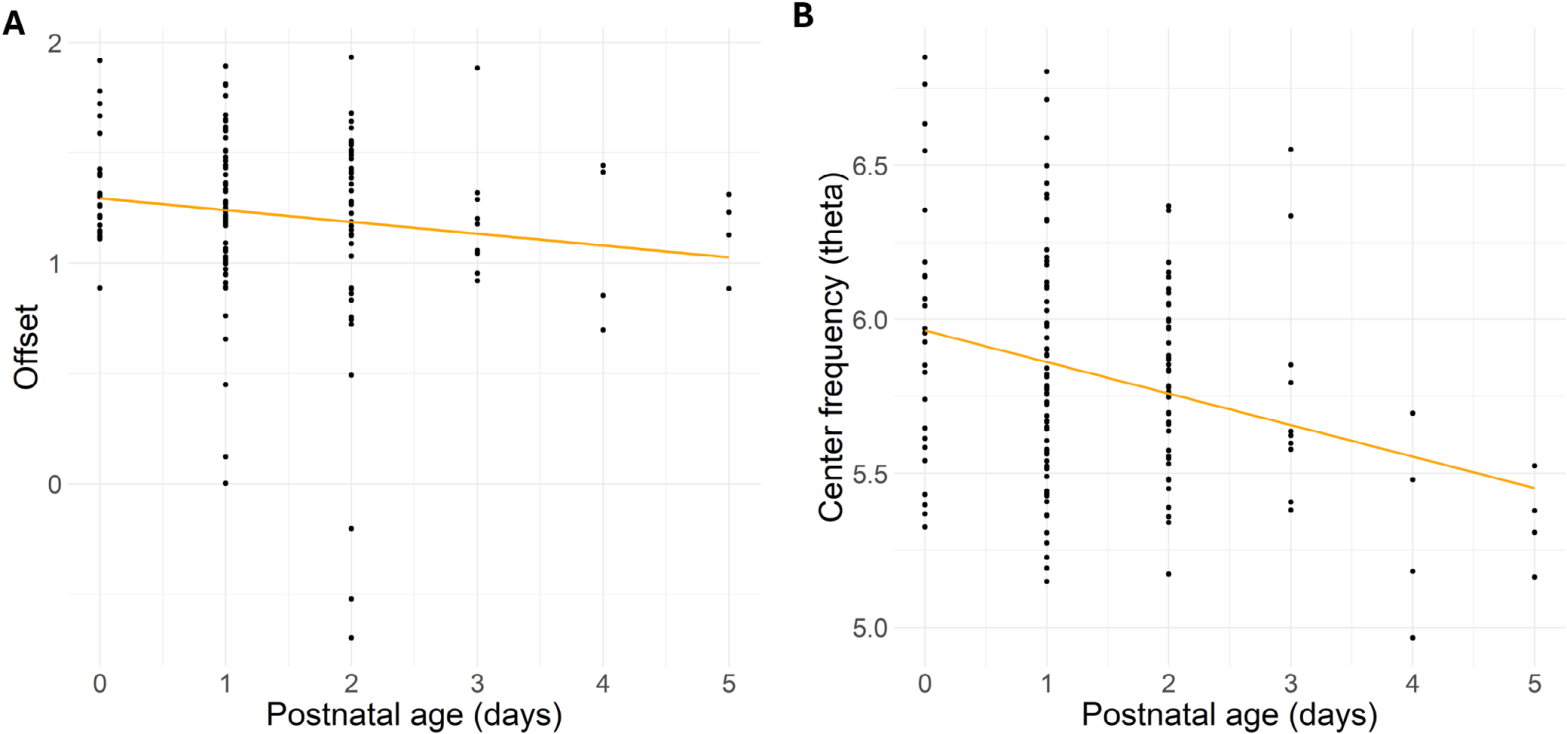
Scatterplot showing the relationship between postnatal age (days) and the predicted offset (A) and theta center frequency (B) values, with a linear regression line fitted to the predicted data points.

We did not find a statistically significant association between offset and gestational duration. However, a statistically significant association was found between the postnatal age of the neonate and the offset (Estimate = −0.07, SE 0.03, *p* = 0.022), reflecting a 0.07 decrease in offset when postnatal age of the neonate increased one standard deviation (SD). Also, a statistically significant association was found between the offset and sleep versus the auditory paradigm + sleep (Estimate = 0.04, SE 0.01, *p* < 0.001), suggesting 0.04 higher offsets during the auditory + sleep versus sleep paradigm. Also, an association between the exponent and the offset was statistically significant (Estimate = 0.34, SE = 0.01, *p* < 0.001). No statistically significant associations were found between other independent variables and offset. For the results, see: Table 3 and Figure 7A.

**TABLE 3:** Results of the multilevel model for offset in neonates (*N* = 73/observations = 144). Residual outliers (*N* = 2) excluded.

	Estimate	SE	DF	*t*	*p*
Intercept	1.22	0.05	70	26.51	**< 0.001**
Gestational duration	−0.03	0.04	67	−0.76	0.448
Postnatal age	−0.07	0.03	67	−2.35	**0.022**
Condition	0.04	0.01	70	3.63	**0.001**
Sex	−0.03	0.06	67	−0.44	0.661
Birth weight	0.01	0.04	67	0.26	0.798
Exponent	0.34	0.01	70	25.03	**< 0.001**
Marginal *R* ^2^/conditional *R* ^2^ of the model					0.643/0.974

*Note:* Statistically significant *p*‐values (< 0.05) are highlighted.

#### 3.1.3 Center Frequency

##### 3.1.3.1 Theta (4–8 Hz)

We did not find a statistically significant association between theta center frequency and gestational duration. The association between postnatal age of the neonate and theta center frequency was statistically significant (Estimate = −0.14, SE = 0.06, *p* = 0.024), as well as the association between birth weight of the neonate and theta center frequency (Estimate = −0.14, SE = 0.07, *p* = 0.043). This means that theta center frequency decreased as a function of the postnatal age as well as the birth weight of the neonate. A statistically significant association between the experimental condition (sleep/auditory paradigm + sleep) and theta center frequency was also observed (Estimate = 0.22, SE = 0.08, *p* = 0.008). This means that during the auditory paradigm + sleep, the theta center frequency was 0.22 higher when compared with the sleep‐alone data, in general. There were no significant associations between center frequency and other independent variables. For the results, see: Table 4 and Figures 6B and 7B.

**TABLE 4:** Results of the multilevel model for center frequency (theta band) in neonates (*N* = 73/observations = 145).

	Estimate	SE	DF	*t*	*p*
Intercept	5.69	0.10	71	58.84	**< 0.001**
Gestational duration	−0.11	0.07	68	−1.51	0.137
Postnatal age	−0.14	0.06	68	−2.30	**0.024**
Condition	0.22	0.08	71	2.74	**0.008**
Sex	0.03	0.12	68	0.25	0.804
Birth weight	−0.14	0.07	68	−2.06	**0.043**
Marginal *R* ^2^/conditional *R* ^2^ of the model					0.172/0.484

*Note:* Statistically significant *p*‐values (< 0.05) are highlighted.

##### 3.1.3.2 Alpha (8–12.5 Hz)

There were not significant associations between alpha center frequency and any of the independent variables.

##### 3.1.3.3 Beta (12.5–30 Hz)

**FIGURE 8:**
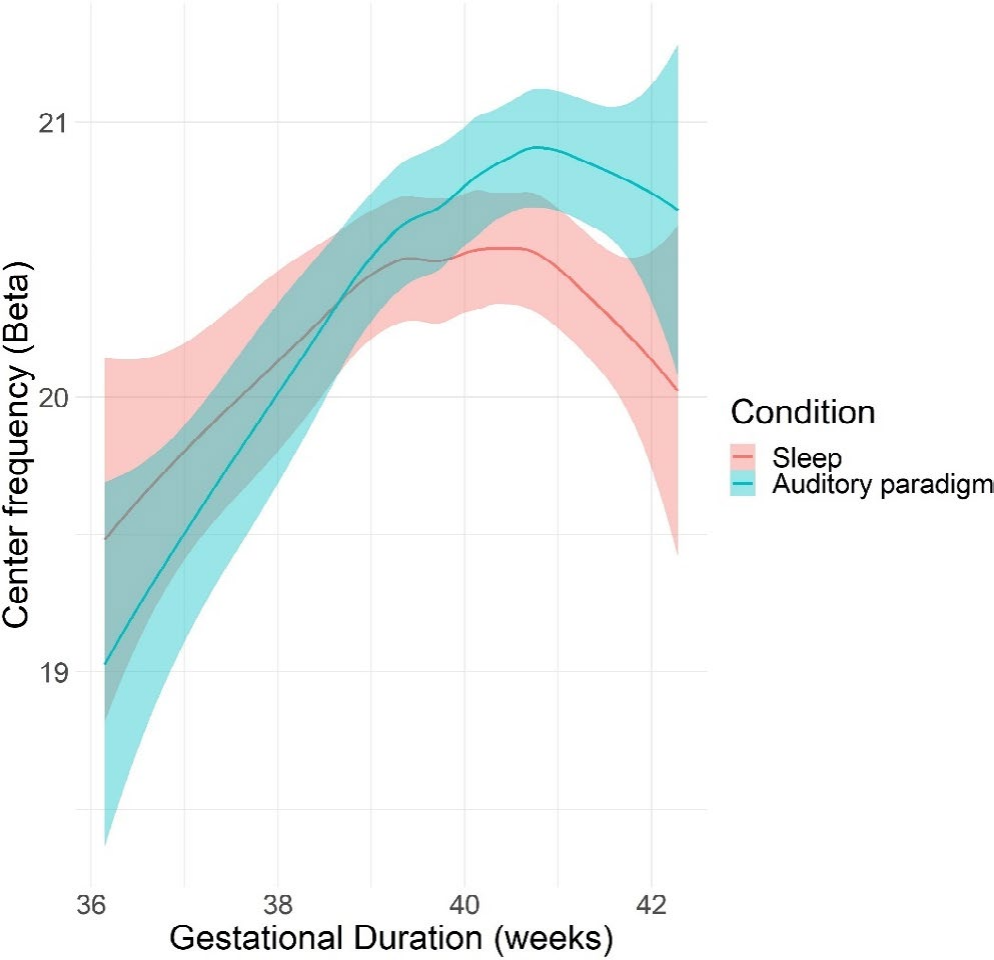
Nonlinear effect of gestational duration on predicted beta center frequency values by condition (auditory paradigm + sleep/sleep) in neonates (*N* = 73). The plot accounts for both the linear and quadratic components of the gestational duration. Shaded ribbons indicate 95% confidence intervals.

The association between gestational duration (quadratic) and exponent was statistically significant (Estimate = −0.14, SE = 0.06, *p* = 0.037), suggesting a nonlinear relationship between gestational duration and beta center frequency. In addition, the interaction effect between gestational duration and condition was borderline statistically significant (Estimate = 0.23, SE = 0.12, *p* = 0.050), which means that the effect of the gestational duration may be different during the auditory paradigm + sleep when compared to the sleep state alone. An association between the experimental condition (sleep/auditory paradigm + sleep) and beta center frequency was also observed (Estimate = 0.25, SE = 0.12, *p* = 0.035). This means that during the auditory paradigm + sleep, the beta center frequency was 0.25 higher when compared with the sleep alone data. For the results, see: Table 5 and Figure 8.

**TABLE 5:** Results of the multilevel model for center frequency (beta band) in neonates (*N* = 73/observations = 146).

	Estimate	SE	DF	*t*	*p*
Intercept	20.45	0.17	71	120.06	**< 0.001**
Gestational duration	−0.13	0.14	67	−0.92	0.361
Gestational duration^2^	−0.14	0.06	67	−2.12	**0.037**
Postnatal age	−0.14	0.10	67	−1.45	0.153
Condition	0.25	0.12	71	2.15	**0.035**
Sex	0.13	0.20	67	0.67	0.504
Birth weight	0.13	0.11	67	1.17	0.248
Gestational duration × condition	0.23	0.12	71	2.00	0.050
Marginal *R* ^2^/conditional *R* ^2^ of the model					0.144/0.528

*Note:* Statistically significant *p*‐values (< 0.05) are highlighted.

#### 3.1.4 Sensitivity Analyses

Sensitivity analyses (maternal BMI/tobacco smoking during the first trimester of pregnancy/postmenstrual age) influenced the results of the theta center frequency model. Specifically, the effect of birth weight (*p* = 0.043) decreased to below statistical significance when tobacco (*p* = 0.065) or maternal pre‐pregnancy BMI (*p* = 0.051) were included in the models. In the beta center frequency model, tobacco altered the statistically significant effects of condition (*p* = 0.079) and the condition × gestational duration interaction (*p* = 0.080). Otherwise, the sensitivity analyses did not affect the statistical significance of the results in any of the models.

### 3.2 Toddlers

#### 3.2.1 Exponent

**FIGURE 9:**
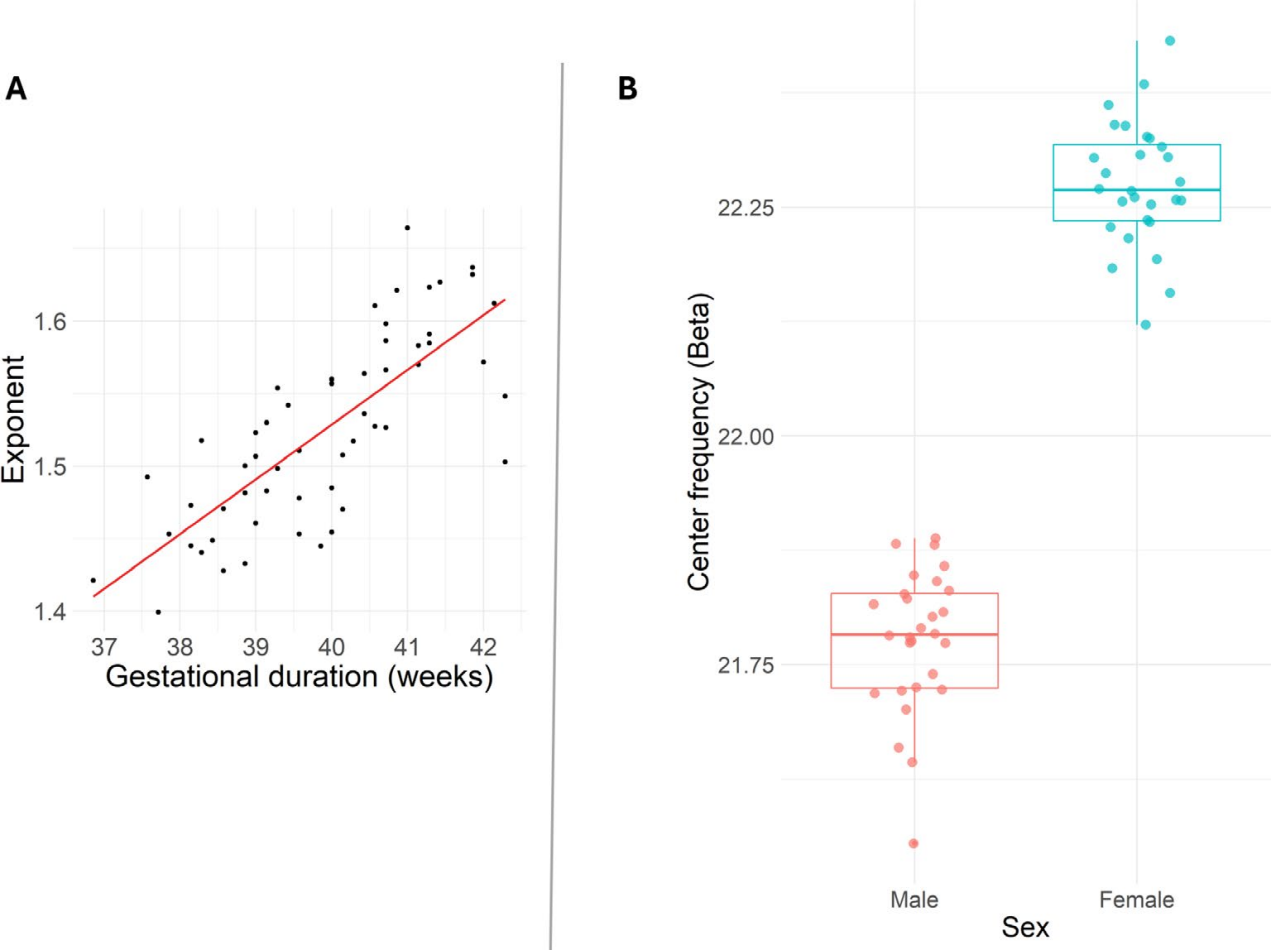
Results of the linear models in toddlers (*N* = 56). (A) Scatterplot of predicted values of the exponent (A) across gestational duration (weeks) from the linear models in toddlers. (B) Distribution of predicted center frequency (beta) values for females and males. Individual predicted values are displayed as points, while the boxplots summarize the distribution within each group. The boxes represent the interquartile range (middle 50% of the data), with the line inside each box indicating the median predicted value. Whiskers extend to the smallest and largest values within 1.5 times the interquartile range, with outliers shown as individual points outside this range.

A statistically significant positive association between the exponent and the gestational duration of the child was observed (*β* = 0.45, SE = 0.02, *p* value = 0.004), suggesting an increase in the exponent as the gestational age of the child increased. In addition, the birth weight of the child and the exponent (*β* = −0.29, SE = 0.02, *p* = 0.056) showed a nearly statistically significant association, suggesting that the exponent decreased as a function of birth weight. Other independent variables did not show any statistically significant associations with the exponent. For the results, see: Table 6 and Figure 9A.

**TABLE 6:** Results of the linear regression model for exponent in toddlers (*N* = 56).

	Estimate	*β*	SE	*t*	*p*	Partial *R* ^2^
Intercept	1.54	NA	0.03	53.20	**< 0.001**	NA
Gestational duration	0.07	0.45	0.02	3.00	**0.004**	0.150
Postnatal age	0.00	−0.02	0.02	−0.18	0.860	0.001
Sex	−0.03	−0.09	0.04	−0.65	0.520	0.008
Birth weight	−0.05	−0.29	0.02	−1.96	0.056	0.070
Adjusted *R* ^2^ of the model						0.101

*Note:* Statistically significant *p*‐values (< 0.05) are highlighted.

#### 3.2.2 Offset

We did not find a statistically significant association between offset and gestational duration. A statistically significant positive association was found between offset and exponent (*β* = 0.85, SE = 0.01, *p* < 0.001). No other statistically significant associations were found.

#### 3.2.3 Center Frequency

##### 3.2.3.1 Theta (4–8 Hz)

There were not significant associations between theta center frequency and any of the independent variables.

##### 3.2.3.2 Alpha (8–12.5 Hz)

There were not significant associations between alpha center frequency and any of the independent variables.

##### 3.2.3.3 Beta (12.5–30 Hz)

A statistically significant association between beta center frequency and the child sex was observed (*β* = 0.35, SE = 0.18, *p* = 0.012), suggesting that beta center frequency values were higher in females compared to males. No other statistically significant associations were found. For the results, see: Table 7 and Figure 8B.

**TABLE 7:** Results of the linear regression model for center frequency (beta band) in toddlers (*N* = 56).

	Estimate	*β*	SE	*t*	*p*	Partial *R* ^2^
Intercept	21.79	NA	0.13	172.85	**< 0.001**	NA
Gestational duration	0.03	0.04	0.10	0.24	0.809	0.001
Postnatal age	0.02	0.02	0.09	0.17	0.865	0.001
Sex	0.47	0.35	0.18	2.61	**0.012**	0.12
Birth weight	−0.08	−0.12	0.10	−0.80	0.427	0.01
Adjusted *R* ^2^ of the model						0.079

*Note:* Statistically significant *p*‐values (< 0.05) are highlighted.

#### 3.2.4 Sensitivity Analyses

Sensitivity analyses (maternal BMI/tobacco smoking during first trimester of pregnancy) did not affect the statistical significance of the results in any of the models.

## 4 Discussion

In this study we demonstrated that gestational duration was positively associated with the aperiodic exponent in neonates and, interestingly, also in toddlers at the age of 3 years. This indicates that the slope of the EEG power spectrum is steeper for those who have higher gestational duration. However, a statistically significant association between offset and gestational duration was not observed, indicating that the uniform shift of power across frequencies would not be related to gestational duration. In toddlers, beta center frequencies were higher in females compared to males. Birth weight was negatively associated with offsets in neonates and toddlers and with theta center frequencies in neonates. To the best of our knowledge, this study represents the first exploration of gestational duration‐related effects on aperiodic and periodic activity in the EEG power spectrum, expanding our understanding of neural activity during early life in typically developing children.

### 4.1 Findings Related to Gestational Duration

Our study revealed a positive association between gestational duration and exponent values in both neonatal and toddler EEG power spectra. While this association was surprisingly strong in toddlers, the effect was weaker in neonates. This indicates that longer gestational duration is associated with a steeper distribution of EEG signal power across frequencies and more prominent low‐frequency fluctuations in the signal. While, to the best of our knowledge, our study is the first to explore the associations between gestational duration and aperiodic activity, it is challenging to interpret these findings in the context of prior research. However, regarding postnatal age, the majority of existing evidence suggests that the slope of the power spectrum flattens with increasing postnatal age across infancy and toddlerhood (Stanyard et al. 2024), but opposite findings have also been reported (Wilkinson et al. 2024a). Based on earlier literature and our findings, it appears that changes in the slope of the power spectrum may differ between the gestation and postnatal periods. We did not find associations between gestational duration with aperiodic offset, which would suggest that the change in power spectrum would be driven more by change in slope of the spectrum than with the uniform shift in power across the frequencies.

The steepness of the power distribution in spectra is proposed to reflect underlying synaptic currents and the balance between excitatory and inhibitory activity (E–I balance). Here, a steeper slope (higher exponent) indicates a shift in this balance from excitatory toward more inhibitory activity, likely influenced by higher gamma‐aminobutyric acid (GABA) synapse density or a shift in GABAergic neurons from excitatory to inhibitory (Voytek and Knight 2015; Gao, Peterson, and Voytek 2017). Noteworthy, this shift may occur due to changes both in excitatory as well as inhibitory activity as shown by Antoine et al. (2019). During the early neurodevelopment, this transition of GABAergic neurons from excitatory to inhibitory is primarily driven by the expression and activity of chloride transporters (McArdle et al. 2024). GABAergic systems undergo rapid maturation during late gestation and early postnatal life, but the idea that it continues to mature postnatally until late adolescence lacks convincing support (Basu et al. 2021; Kilb 2012). Changes in power spectral densities reflect the aggregate activity of extracellular voltage deflections (local field potentials), which are influenced by, for example, slower‐decaying inhibitory GABA currents and faster‐decaying excitatory AMPA currents. This combined activity can be measured noninvasively using EEG (Buzsáki, Anastassiou, and Koch 2012). Our results suggest that the steepening slope of the power spectrum could serve as a marker for neurodevelopmental processes during gestation, potentially reflecting the rapid synaptogenesis occurring in the developing brain.

While development‐related changes in power spectrum slope have been widely interpreted as changes in neuronal activity in prior studies (Stanyard et al. 2024), recent work by Schmidt et al. (2024) showed that aperiodic parameters are also influenced by non‐neural physiological factors (e.g., ECG, electrocardiogram) in adults. Similar associations between ECG aperiodic activity and the EEG power spectrum have not yet been studied in younger children or adolescents, and this should be addressed in future research.

Our finding that the association between a steeper slope of the power spectrum and gestational duration is stronger in toddlers compared to neonates is noteworthy and deserves further attention. While this difference could, for example, be due to a lower signal‐to‐noise ratio in the neonate datasets compared to the toddler dataset, it is also possible that the effect of gestational duration becomes more pronounced during the early years of life. It is important to note that our participants were primarily term‐born or early‐term born children (gestational age > 36 weeks). Our results suggest that even relatively small changes in gestational duration may have significant effects on brain physiology.

Given that alterations in the slope of the power spectrum have been implicated in conditions such as attention deficit hyperactivity disorder (ADHD) (Karalunas et al. 2022; Robertson et al. 2019) and autistic traits (Carter Leno et al. 2022)—both of which are more prevalent in preterm‐born children (Peralta‐Carcelen, Schwartz, and Carcelen 2018)—future research could explore the potential mediating role of the slope of the power spectrum in these relationships. Prior research indicates that even early‐term children are at risk for poorer long‐term developmental outcomes, including school performance, neurodevelopment, behavior, emotional status, and social outcomes (Dong, Chen, and Yu 2012). Conversely, longer gestational durations in full‐term children may be beneficial for early mental and motor development (Espel et al. 2014). Investigating the potential role of the aperiodic slope in predicting other outcomes related to gestational duration, such as cognitive ability, holds promise for further understanding these relationships.

In addition to associations with exponent, we also observed a quadratic association between beta center frequencies and gestational duration in neonates, with a positive association until around gestational week 40–41, after which the association seemed to turn negative. Also, the interaction between condition (sleep/auditory paradigm + sleep) and gestational duration was statistically significant, with children born after a gestational duration of 38 weeks showing higher beta frequencies during the auditory paradigm + sleep when compared to sleep state alone.

The quadratic effect of gestational duration may be explained by a shift in neural activity during late gestation, from discrete, spontaneous, and transient activity to more continuous high‐frequency activity, which is essential for most cognitive functions (Vasung et al. 2019). Additionally, brain growth is rapid in late gestation, with brain volume increasing nearly three‐fold and grey matter constituting up to 50% of total brain volume between gestational weeks 29 and 41 (Hüppi et al. 1998). We propose that this rapid growth could be reflected as higher beta frequencies. The decrease in beta frequencies after a gestational duration of 41 weeks may be attributed to the onset of synaptic pruning, occurring already in late gestation (Buzsáki, Anastassiou, and Koch 2012). Moreover, sleep state changes occur toward late gestation, with the percentage of time spent in active sleep increasing until gestational weeks 39–41 (Bourel‐Ponchel et al. 2021). However, by the end of gestation and into early postnatal life, the amount of quiet sleep increases relative to active sleep (Louis et al. 1997; Ficca, Fagioli, and Salzarulo 2000; Coons and Guilleminault 1982). Since beta oscillations are more prominent during active sleep compared with quiet sleep in neonates (Peralta‐Carcelen, Schwartz, and Carcelen 2018), this change could explain the nonlinear association between gestational duration and beta frequencies we observed. The developmental origins of beta frequencies, particularly during the fetal and neonate period, require further investigation.

### 4.2 Findings Related to Postnatal Age

We observed statistically significant associations between postnatal age and offset as well as theta center frequency in neonates, but not in toddlers. Our findings suggest a reduction in the baseline power of the signal across frequencies and an age‐related progression of theta oscillations toward lower frequencies during the first days after birth.

Our observation that offset decreased as a function of postnatal age contradicts the results of a study by Wilkinson et al. (2024a), which reported an increase in aperiodic offset and exponent during the first year of life. In their study, the offset increase was especially rapid during the first year of life, with only minimal changes between the ages of 1 and 3 years. This pattern likely reflects the known increases in brain volume and synaptogenesis during the neonatal period and infancy (Knickmeyer et al. 2008; Bethlehem et al. 2022; Tau and Peterson 2010).

The transition from a low‐sensory intrauterine to a sensory‐rich extrauterine environment requires rapid adaptation of multiple organ systems, including the nervous system (Morton and Brodsky 2016). During the mid‐fetal to early preterm period, EEG is more discontinuous, showing desynchronized patterns and patterns of discrete large spontaneous activity transient waves, while around gestational week 42, circuits with sensory‐driven activity develop, when cortico‐cortical axons establish synapses with pyramidal neurons (Vasung et al. 2019). We suggest that this shift from more spontaneous activity toward sensory‐driven activity reduces baseline aperiodic activity, reflected as a decrease in aperiodic offset. Additionally, the sleep architecture of neonates becomes more structured after birth, as quiet sleep, characterized by slower oscillations, begins to dominate over active sleep (Louis et al. 1997; Ficca, Fagioli, and Salzarulo 2000; Coons and Guilleminault 1982). We propose that the observed decreases in offset and theta center frequency may be caused by a combination of functional adaptations to the extrauterine environment and maturation‐related changes in sleep architecture.

Interestingly, we did not find any statistically significant associations between aperiodic exponent and postnatal age, despite several previous studies reporting such associations during the first years of life (Stanyard et al. 2024). It is important to note that our study was not longitudinal, and the lack of statistically significant associations between postnatal age and aperiodic exponent may be attributed to the small variation in postnatal age among toddlers (range 34.8–38.2 months) and neonates (range 0–5 days).

### 4.3 Other Factors Contributing to Aperiodic Parameters and Center Frequency

#### 4.3.1 Sex

In toddlers, females showed higher beta center frequencies compared to males in our study. Beta frequencies are traditionally associated with sensorimotor processing and have since been linked to a broader range of cognitive functions, such as working memory and decision making (Spitzer and Haegens 2017). The origins of beta oscillations are assumed to be at the cortical network level, facilitating long‐range interactions and linked to top‐down controlled processing (Spitzer and Haegens 2017). Findings from a prior study by Wilkinson et al. (2024a) suggest that during early development, beta peaks can be divided into lower (12–20 Hz) and higher (20–30 Hz) peaks, with potentially distinct developmental origins. However, the higher beta peak appeared to be transient, as by the age of 36 months, the low beta peak became dominant again. Their study also reported a parallel trend of females exhibiting higher beta peak frequencies compared to males across the ages of 0–1200 days (Wilkinson et al. 2024a).

Our findings provide further support for sex‐related differences in child electrophysiological activity during toddlerhood, but not during the neonatal period. This discrepancy may reflect more mature cortical‐level neural networks in females compared to males during this developmental stage. To better understand these differences, longitudinal studies across early life are required in the future.

#### 4.3.2 Birth Weight

Birth weight was negatively associated with exponent in neonates and toddlers, but in toddlers, the association was only nearly statistically significant. In addition, birth weight was negatively associated with theta center frequencies in neonates, but not in toddlers. Our findings indicate a flatter distribution of power across frequencies in children with higher birth weight and the progression of theta oscillation toward lower frequencies in neonates as a function of birth weight.

Existing literature predominantly focuses on the effects of birth weight in very low or extremely low birth weight individuals, often born prematurely (Ment et al. 2009; Kuula et al. 2022; Hayward et al. 2020). For instance, a prior study has demonstrated that extremely low birth weight adults (without any impairments) show more spectral power in low EEG frequency bands and less in high‐frequency bands compared to their normal birth weight peers (Miskovic et al. 2009). As our study participants were born with normal birth weight, a direct comparison with prior findings is challenging. Our results suggest that even within the range of normal birth weights, differences could have potentially long‐term associations with neural activity in later life.

The influence of normal birth weight on later neurodevelopment might be elucidated through fetal programming, as proposed by the Developmental Origins of Health and Disease (DOHaD) hypothesis (Barker 1998). It suggests that exposure to adverse environmental experiences during critical periods of early development may permanently alter the cellular, organ, and physiological system structures and functions (Barker 1998). Proposed main mechanisms of fetal programming include fetal malnutrition and overexposure to glucocorticoids (Harris and Seckl 2011; Räikkönen et al. 2011; Seckl and Holmes 2007), but also epigenetic mechanisms (i.e., molecular mechanisms altering gene expression) in mediating the relationship between fetal development and later health are suggested (Doi, Usui, and Shimada 2022). Thus, prenatal nutrition and birth weight may interact with genetic and environmental factors to modulate neurodevelopment, with consequences that can be observed during the later postnatal development.

The decrease in theta frequency as a function of birth weight could also be explained by differences in brain size, as children with greater birth weight have also been shown to have larger brain volumes (Wang et al. 2018), which could potentially result in slower signal progression across networks.

Finally, considering that prematurity and low birth weight often, but not always, occur together (Stein, Siegel, and Bauman 2006), it is interesting that our findings from birth weight contradict those observed with gestational duration. As current research in this area is limited, the interpretation of our results is challenging, and we are unable to delve further into this question. Further investigation is required to explore the effects of birth weight, particularly in individuals born with normal birth weight, on subsequent developmental and health outcomes.

### 4.4 Limitations

In discussing the methodology and limitations of our study, several key points merit attention.

First, it is important to note that our study employed a cross‐sectional design, which limits our ability to infer changes in neural activity over time in individuals. However, we were able to gather data from two different age groups, despite the scarcity of prior EEG studies in toddlers, primarily due to challenges in behavioral measures (Putkinen et al. 2012). Additionally, it is worth mentioning that studies focusing on aperiodic activity in neonates and infants are rare, and some of them have utilized smaller sample sizes than ours (Schaworonkow and Voytek 2021).

Second, the lack of information regarding the sleep state of neonates (active/quiet sleep) may complicate the interpretation of our results, as aperiodic parameters differ between wakefulness and sleep in children (Favaro et al. 2023). Sleep–wake patterns among newborns undergo rapid evolution during the first few months after birth, transitioning from relatively active to structured non‐rapid eye movement and rapid eye movement sleep phases (Louis et al. 1997; Ficca, Fagioli, and Salzarulo 2000; Coons and Guilleminault 1982). Given the challenges in distinguishing sleep states in this age group, we consciously attribute observed changes in aperiodic parameters to factors such as gestational duration and aging of the neonates.

Third, our reliance on data collected during auditory stimulation to estimate power spectra may present some limitations. While prior electrophysiological studies have typically utilized resting‐state data (Hill et al. 2022), our neonate model revealed that offsets and theta center frequencies were higher in the auditory paradigm + sleep data compared to sleep alone data. Additionally, the absolute errors of SpecParam fits were generally higher in the theta/alpha ranges (~5–15 Hz), particularly in the toddler dataset. This may reflect increased variance in the dataset due to individual responses to auditory stimuli. During model selection, we observed condition‐related (sleep/auditory paradigm + sleep) interaction only with gestational duration in the beta frequency model for neonates, supporting the generalizability of our gestational age‐related group‐level findings. We think our main findings are thus robust, but there is a clear need to study how the presented stimuli might affect the error profiles of SpecParam fits. Our data set did not allow us to address this further. However, further studies with larger sample sizes (e.g., The HEALthy Brain and Child Development Study [Volkow et al. 2024]) could explore the effects of stimuli versus resting‐state conditions on SpecParam fits.

Fourth, it is important to note that while neonates were asleep during the EEG recordings, toddlers were recorded in an awake state. Given that the neonates were measured at only 0–5 days of postnatal age, sleep was the only reliable way to obtain data from this challenging age group. Although we could not avoid the issue of differing alert states between the two age groups, we assume that the observed differences are likely driven by factors such as postnatal maturation. Caution is required when interpreting these results.

Fifth, it is essential to note that our samples included children born at or after 36 weeks of gestation, encompassing both late preterm children (born at gestational week 36) and term children (born at gestational week 37 or later). This decision was made as excluding late preterm children would have significantly reduced our sample sizes, limiting the possibilities of statistical modeling. While these age groups are often treated differently in research, gestational age‐related effects appear to be more of a continuum (Engle 2011), providing some confidence that our results could be generalized to a larger population.

## 5 Conclusion

Our results showed, for the first time, that gestational duration is associated with aperiodic activity, suggesting the steeper distribution of spectrum power across the frequencies in neonates and toddlers with longer gestational duration. Interestingly, the association was even stronger in toddlers than in neonates, suggesting that gestational duration may have significant and relatively long‐lasting effects on brain physiology. The possible behavioral and cognitive consequences of these changes remain to be elucidated in future research.

## Supporting information


**Data S1.** Supporting Information.

## Data Availability

The datasets presented in this article are not readily available because for now, the data cannot be shared openly due to Finnish legislation and our ethical permission, but data can be made available through formal data sharing/material transfer agreements. Requests to access the datasets should be directed to FinnBrain administration (https://sites.utu.fi/finnbrain/en/contact/).
