# Supplementary material for: Gestational Duration and Postnatal Age‐Related Changes in Aperiodic and Periodic Parameters in Neonatal and Toddler Electroencephalogram (EEG)
Source: Hum Brain Mapp. 2025 Jan 7;46(1):e70130. doi: 10.1002/hbm.70130 (PMC11705402; doi:10.1002/hbm.70130)
Supplement: Supplementary file 1 — Data S1. Supporting Information. [file HBM-46-e70130-s001.docx]

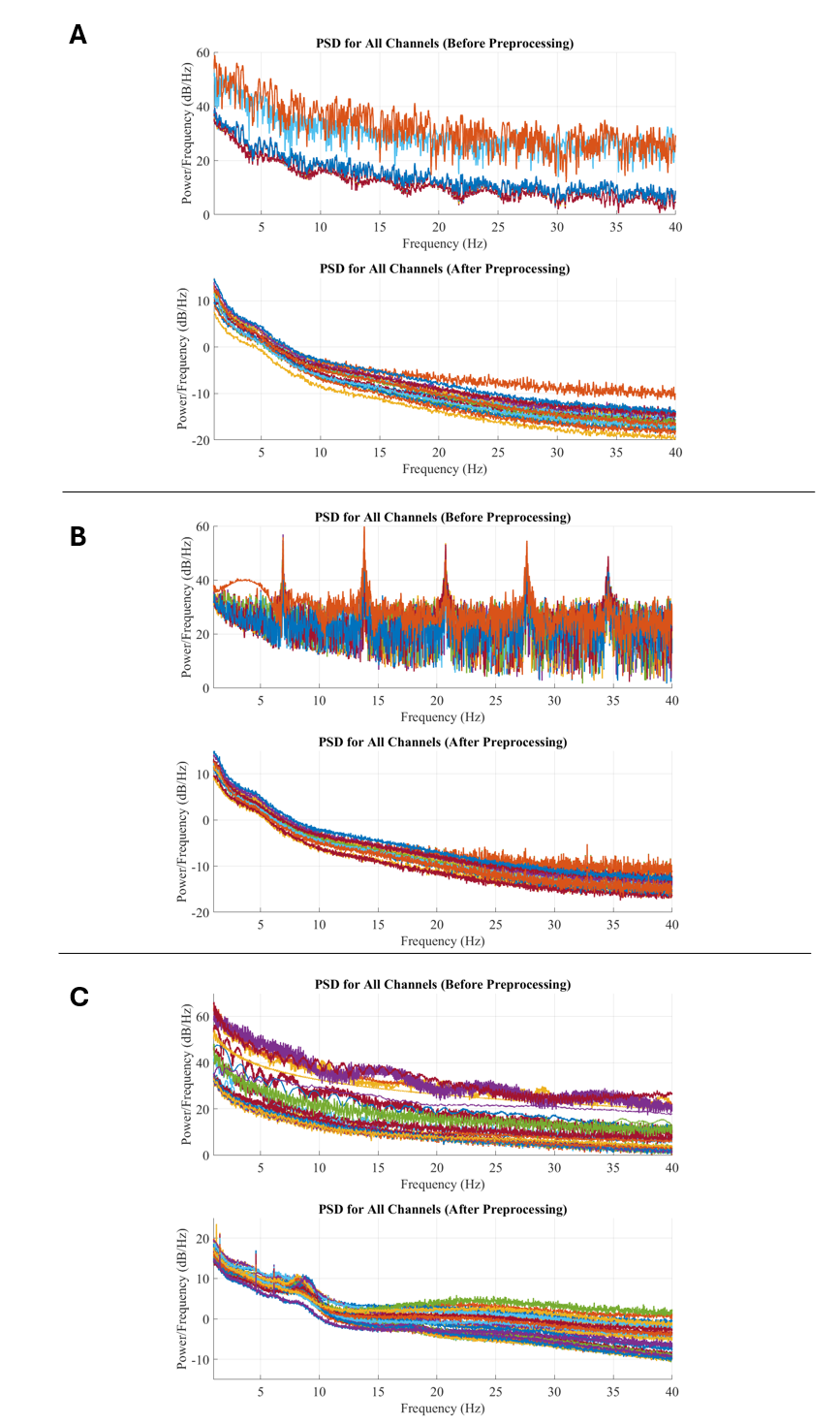


**Supplemental Figure 1.** Power spectra before and after the preprocessing (Artifact Subspace Reconstruction (ASR) and interpolation of the channels) of each data set averaged across participants. Each channel presented separately (16 channels in neonates, 32 channels in toddlers). A) Neonate / sleep (N = 73). B) Neonate / auditory paradigm (N = 73). C) Toddler / auditory paradigm (N = 56).

| **Supplemental Table 1**. Mean / SD / Range of interpolated electrodes in each data set. | | | | |
| --- | --- | --- | --- | --- |
|  | Number of channels (total) | Interpolated channels | | |
| Data set |  | Mean | SD | Range |
| Neonate / Sleep (N = 73) | 16 | 4.18 | 1.77 | 1–9 |
| Neonate / Auditory paradigm (N = 73) | 16 | 4.56 | 1.67 | 2–10 |
| Toddlers (N = 56) | 32 | 4.70 | 2.66 | 1-15 |


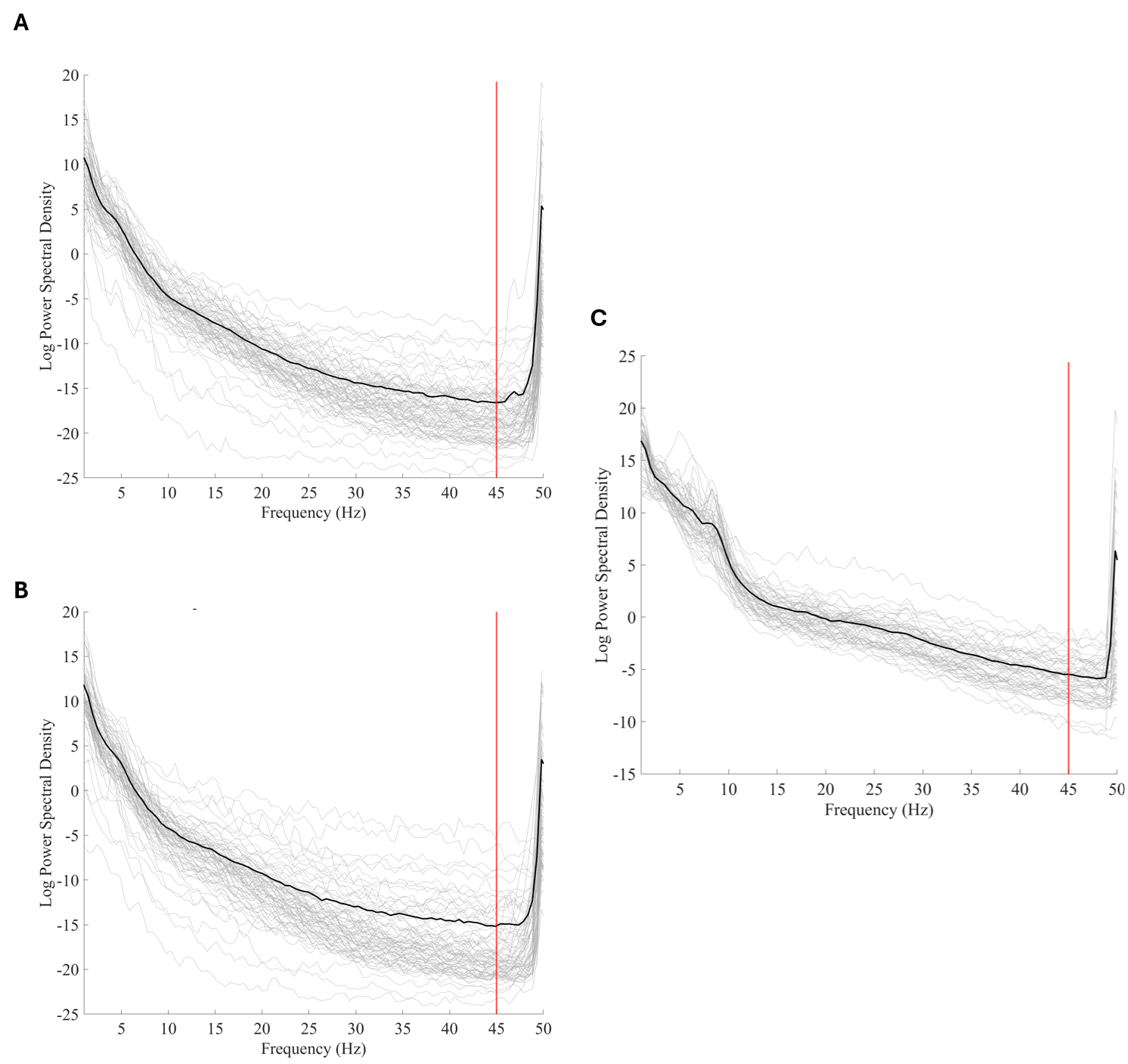


**Supplemental Figure 2.** Individual power spectra and mean power spectra from 0Hz to 50Hz in each dataset. Red line stands for upper boundary for SpecParam frequency range (45Hz). Channels with poor-fit segments (FOOOF fit R^2^ < 0.95) excluded.

| **Supplemental Table 2**. Mean / SD / Range of SpecParam fit R^2^ ‘s, number of segments and number of poor-fit segments / channel in each data sets. | | | | |
| --- | --- | --- | --- | --- |
| Data set | Parameter | Mean | SD | Range |
| Neonate / Sleep  N = 73 | Mean R^2^ of SpecParam (averaged over segments and channels, poor-quality segments excluded) | 0.99 | 0.00 | 0.98–1.00 |
|  | Total segments (N) | 6.22 | 0.96 | 2–9 |
|  | Mean N of poor-fit (SpecParam R^2^ < 0.95) segments / channel | 0.01 | 0.02 | 0–0.16 |
| Neonate / Auditory paradigm  N = 73 | Mean R^2^ of SpecParam (averaged over segments and channels, poor-quality segments excluded) | 0.99 | 0.00 | 0.97–1.00 |
|  | Total segments (N) | 20.26 | 2.88 | 17–30 |
|  | Mean N of poor-fit (SpecParam R^2^ < 0.95) segments / channel | 0.01 | 0.03 | 0–0.21 |
| Toddlers  N = 56 | Mean R^2^ of SpecParam (averaged over segments and channels, poor-quality segments excluded) | 0.99 | 0.00 | 0.98–0.99 |
|  | Total segments (N) | 18.86 | 3.46 | 9–24 |
|  | Mean N of poor-fit (SpecParam R^2 < 0.95) segments / channel | 0.06 | 0.07 | 0–0.33 |


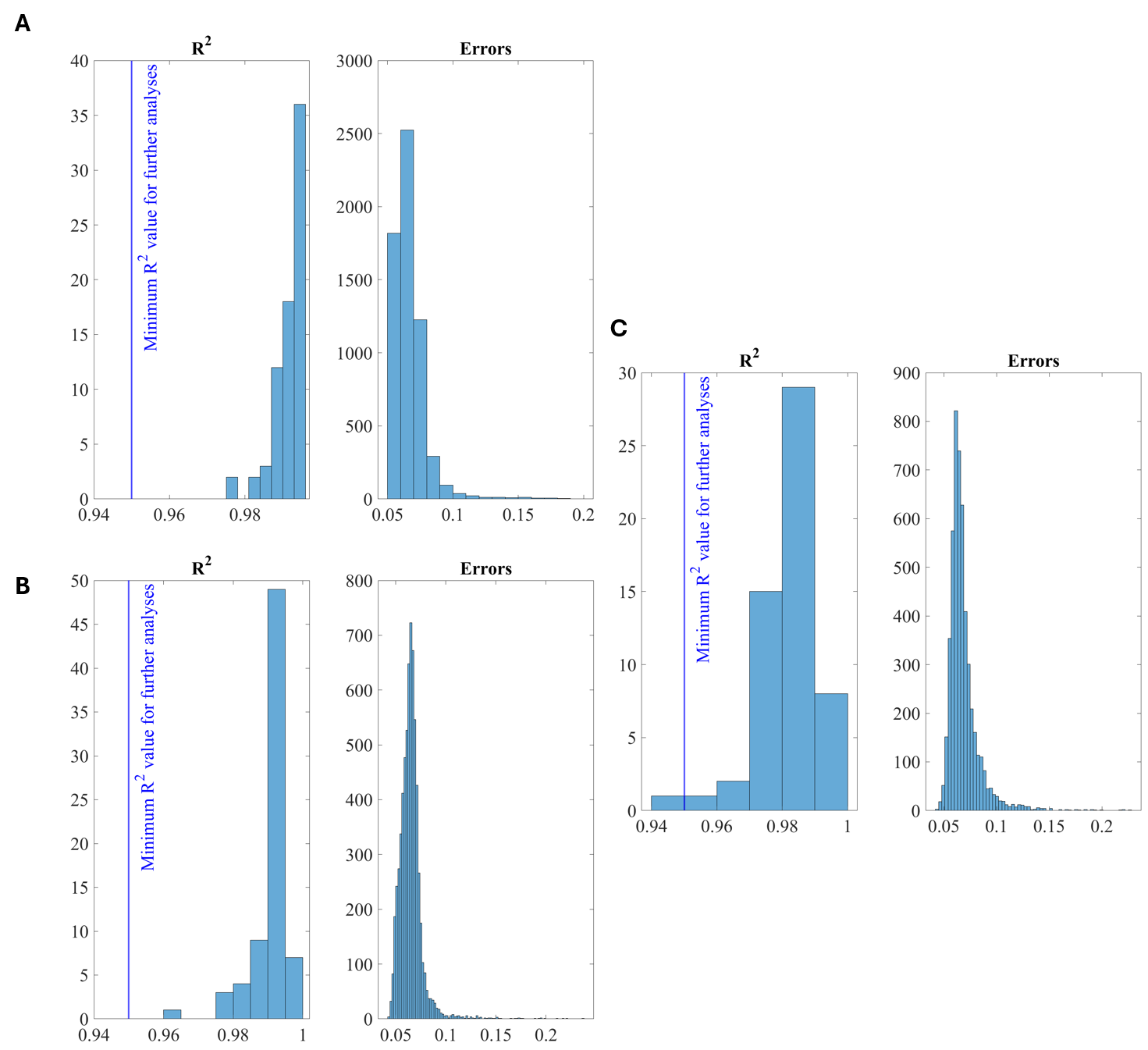


**Supplemental Figure 3.** Histograms of R2’s and errors from SpecParam model output. Values are averaged over segments and channels. A) Neonate sleep (N = 73). B) Neonate Auditory paradigm (N = 73). C) Toddlers (N = 56).


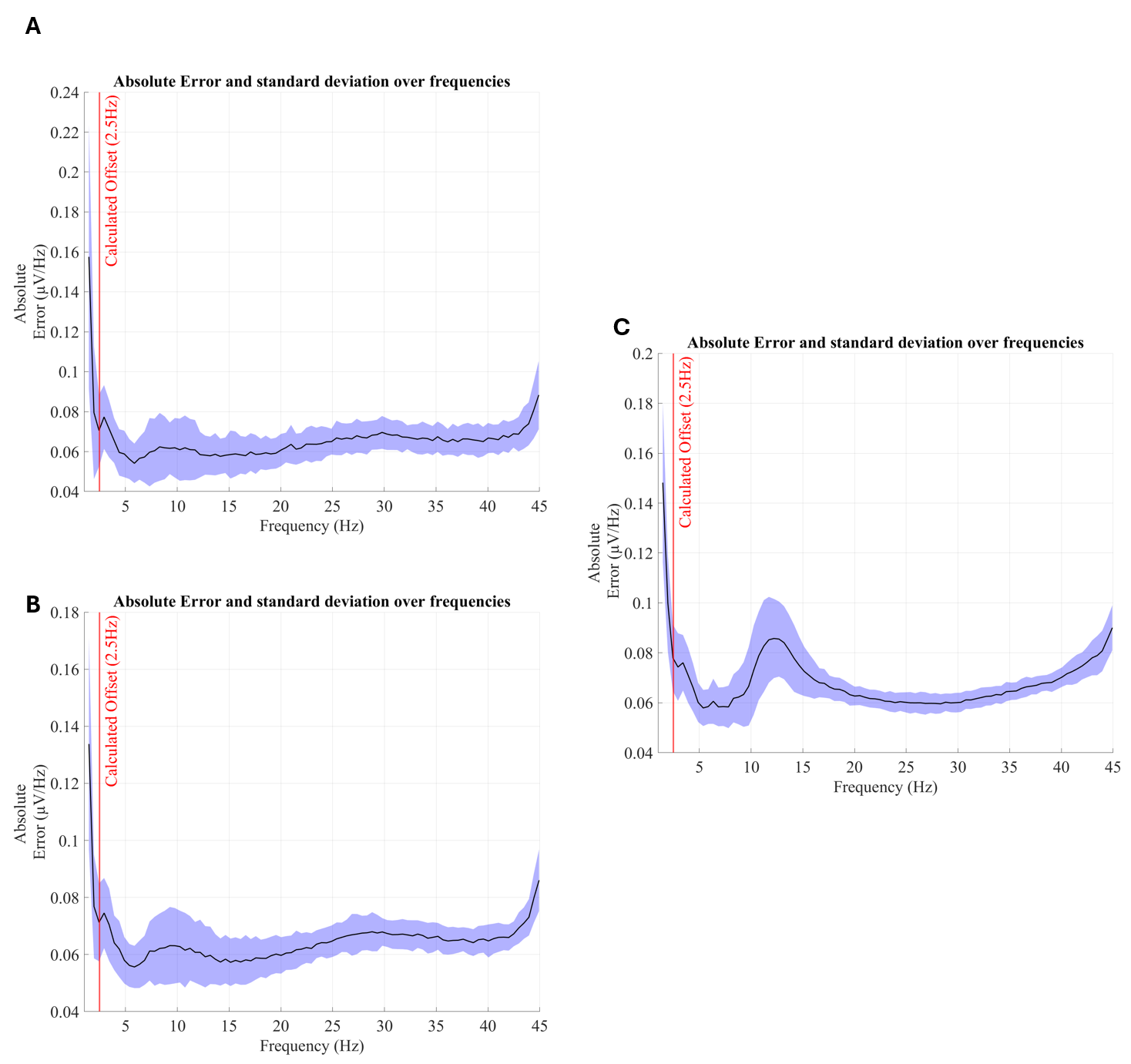


**Supplemental Figure 4.** Mean absolute error from SpecParam per frequency, as well as standard deviation in error per frequency (blue shading). A) Neonate sleep (N = 73). B) Neonate Auditory paradigm (N = 73). C) Toddlers (N = 56).
